# Supplementary material for: Maps and metrics of insecticide-treated net access, use, and nets-per-capita in Africa from 2000-2020
Source: Nat Commun. 2021 Jun 11;12:3589. doi: 10.1038/s41467-021-23707-7 (PMC8196080; doi:10.1038/s41467-021-23707-7)
Supplement: Supplementary file 1 — Supplementary Information [file 41467_2021_23707_MOESM1_ESM.pdf]

Supplementary Information for "Maps and metrics of insecticide-treated net access, use, and nets-per-capita in Africa from 2000-2020"

1 Supplementary Note 1: Stock and Flow Methods

1.1 Background

The goal of the stock and flow model is to estimate insecticide-treated net (ITN) crop and access over time at the national level from 2000 to 2020 for 40 sub-Saharan African countries. The model triangulates and ensures consistency between three data sources: reports from net manufacturers about how many ITNs were delivered to national programs, reports from national programs about how many ITNs were distributed to households, and data extracted from household surveys about how many ITNs were found in houses. The first two data sources are annual; the third is cross-sectional and was observed between one and ten times in each country across the time period of interest.

The difficulty of estimating ITN crop from surveys alone, and the utility of a mechanistic model for incorporating added information from delivery and distribution data, was first introduced by Flaxman et al. in 2010 [1]. This paper established the fundamental framework for a "stock and flow" model of tracking net crop in a given country over time, but was constrained by several limitations. First, net crop was estimated with an annual time step, diluting the 3-4 month time span captured by any nationally representative survey and yielding relatively coarse estimates of net crop over time. Second, rates of net loss were estimated separately in the first, second, and third years of net life, with all nets presumed discarded by the end of three years – a parameterization which makes description of net retention difficult and which may ignore long-term net ownership. Third, the model reported "coverage" in units of "household ownership of at least one

ITN" and "ITN use in children under 5", neither of which are currently recommended as evaluation metrics (though both are still often reported and used for policy decisions).

In 2015, Bhatt et al. [2] published an improved version of the stock and flow model featuring a quarterly time step, a smooth-compact loss function for ITN retention, and the explicit calculation of five different ITN metrics, including household ownership, household access, population access, population use, and the "ownership gap". Limitations of this model included unrealistically high coverage estimates in countries with no available survey data (most notably South Sudan). Additionally, while the decisions to use splines for missing net distribution data imputation and fit two time-varying parameters for the net loss function added an immense amount of flexibility to the model, it concurrently called into question the identifiability of the parameters being fit.

Neither the Flaxman nor Bhatt models reported on several quality assurance metrics that are now standard. First, although the number of surveys available for model fitting differs drastically from country to country (from zero to five in Flaxman's models and zero to nine in Bhatt's), the sensitivity of model fit to survey inclusion is not reported with either model. Second, while both models published extensive documentation, neither group published their code or otherwise made the details of their analysis available for external review or replication.

The present analysis substantially updates and refines the Bhatt model, including:

- Simplified imputation and loss function parameterization to improve identifiability;
- Sensitivity analysis on survey inclusion (Supplement 1.6);
- Updated survey data across the full time series, including previously overlooked surveys from Ethiopia, South Sudan, and Zimbabwe;
- A fully refactored, publicly available codebase with Google Cloud functionality (`https://github.com/bertozzivill/map-itn-cube/tree/publication-2021`).

The only other mechanistic model of national-level ITN crop known to the authors is NetCALC (`https://www.vector-works.org/resources/netcalc-planning-tool/`), a publicly-available spreadsheet-based model designed to assist countries with net procurement calculations. NetCALC is widely used by national programs and net

durability studies [3–6], but is not designed for the type of historical analysis or spatial disaggregation required here.

1.2 Definitions: ITN, LLIN, cITN, Access, Household Size

1.2.1 Net Types

Pre-treated nets whose insecticide is designed to last for at least three years are defined as long-lasting insecticide-treated nets" ("LLINs"). Treated nets obtained, or re-soaked with insecticide, within the past 12 months are defined as conventional ITNs, or "cITNs". LLINs and cITNs collectively comprise "ITNs", or simply "nets", as untreated nets do not enter into this analysis.

1.2.2 Net Movement

ITN "delivery" refers to manufacturer shipment of nets to national programs or other distributing bodies, while ITN "distribution" refers to the provision of nets to homes. Most countries have continuous ITN distribution channels through antenatal clinics, child immunization programs, and schools, which are supplemented every 3-4 years with mass distribution campaigns directly to family homes. ITN "stock" refers to the number of nets available to distribute at a given time. Because national programs may not immediately distribute all of the nets delivered to them, stock in a given year is not necessarily equal to the manufacturer delivery count.

ITN "crop" refers to the total number of nets in homes in a country at a given time point. Crop depends upon both ITN distribution and ITN "retention", the length of time for which nets are owned before being discarded. The WHO recommends mass net distributions every three years under the assumption that average retention times are not much shorter than this.

1.2.3 Household Size

For surveys with data available at the household level, "household size" refers to the number of people who slept in the household the night prior to the survey. For the 33 surveys (Supplementary Table 3.1) for which only aggregated measures

were available, "mean household size" references the survey-specific definition of the term.

1.2.4 Net Access

A person is defined to have "access" to an ITN if they live in a household where they can sleep under an ITN, assuming two people per net per night. Population-level access is the fraction of people with access. To avoid underestimating this metric, access is calculated at the individual level– i.e. in a household of ten people and three nets, six people will be defined as "having access" even though the household as a unit does not have sufficient access for all its inhabitants. Access is calculated from surveys that either count the number of nets in homes or that ask household heads how many nets they own.

1.3 Data sources

1.3.1 Net Stock: LLIN Manufacturer Reports

Net stock data were obtained from the Alliance for Malaria Prevention's (AMP) Net Mapping Project (https://netmappingproject.allianceformalariaprevention.com/) via WHO (private correspondence), and represent the number of LLINs delivered by manufacturers to countries annually from 2000 to 2019. This source includes LLINs donated by Global Fund, the President's Malaria Initiative (PMI), UNICEF, the Against Malaria Foundation (AMF), World Bank, UNITAID, the UK Department for International Development (DfID), the Canadian International Development Agency, private sales, and other sources. These data come directly to AMP from all WHO-approved net manufacturers, and were thus assumed to be highly complete. Data were not available for LLINs in 2020 and do not include cITNs at all, so for these cases the number of nets delivered was assumed to equal the number of nets distributed (see below).

1.3.2 Net Distribution: National Malaria Control Programs, the African Leaders Malaria Alliance, and the President's Malaria Initiative Malaria Operational Plans

The following data sources were available for ITN distributions at the national level:

- National Malaria Control Programs (NMCPs), via WHO: Distribution numbers, disaggregated by net type, from 2000 to 2018, with extensive missing values.
- African Leaders Malaria Alliance (ALMA, https://alma2030.org/): Mass net distribution numbers from 2016 to 2020. These data contain missing values in country-years without mass campaigns. The 2020 distribution numbers were obtained in late September of 2020, and enumerate both nets already delivered and those projected to be delivered before the end of the year. Both of these channels were included in the model as "delivered".
- President's Malaria Initiative Malaria Operational Plans (PMI MOPs, https://www.pmi.gov/resource-library/mops/fy-2020): Twenty-four countries in this analysis receive funding from PMI, and therefore publish annual Malaria Operational Plans (MOPs) which include a three-year assessment of retrospective and prospective ITN need and distribution. For example, the 2019 Tanzanian MOP contains ITN distribution counts for 2018, and proposed distributions for 2019, and 2020. Some reports are later supplemented with a revised funding table giving updated distribution estimates. While these reports are generated by PMI, they include estimates of net distributions from other large in-country donors. These are a less accurate resource than NMCP or ALMA data as they are largely prospective, but provide useful information in the absence of other data.

To create a single cohesive time series of net distributions from 2000 to 2019, data from these sources were combined as follows (Supplementary Fig. 1.1):

1. From 2000 to 2015, NMCP data were used, with the process of missing value imputation described below.
2. From 2016 to 2020, where available, the higher of the NMCP or ALMA values were used.

3. From 2016 to 2020, where neither NMCP nor ALMA data were available but a PMI MOP was present, the latest available PMI estimate was used.

4. If no data from any source was available in a country-year, the minimum NMCP distribution value from 2014-2018 was used. This value was selected as a proxy for a typical number of routinely-distributed nets in a country-year.

NMCP data prior to 2016 were incomplete and required imputation. Missing values were primarily in country-years where the expected number of nets was zero (i.e. for cITNs after 2013 or LLINs prior to 2007), but some countries had missing values at points in the timeseries when nonzero distributions would be expected.

For years prior to each country's mass adoption of LLINs or all years for cITNs, missing distribution counts were set to zero. For missing values in other years, the following case-specific strategies were used to estimate a typical number of routinely distributed nets, avoiding mass distributions:

- Chad 2012: Take the minimum of 2010-2013.
- Cote d'Ivoire 2007-8: Interpolate between 2006 and 2009.
- Cote d'Ivoire 2012: Take the minimum of 2010-2013.
- DRC 2005: Interpolate between 2004 and 2006.
- Mauritania 2007: Interpolate between 2006 and 2008.
- Tanzania 2013-2016: Take values from the most recent available PMI MOPs.
- Togo 2009: Take the minimum of 2008-2011.

Net distribution counts summarize complex campaigns requiring the coordinated effort of thousands of health workers. As reflected in the variety and missingness of the available data, determining and reporting these values presents numerous logistical challenges. The values presented here may not reflect nets lost in the process of distribution, or nets acquired through the private market. To reflect this uncertainty, wide priors were placed on ITN distribution counts in the model.

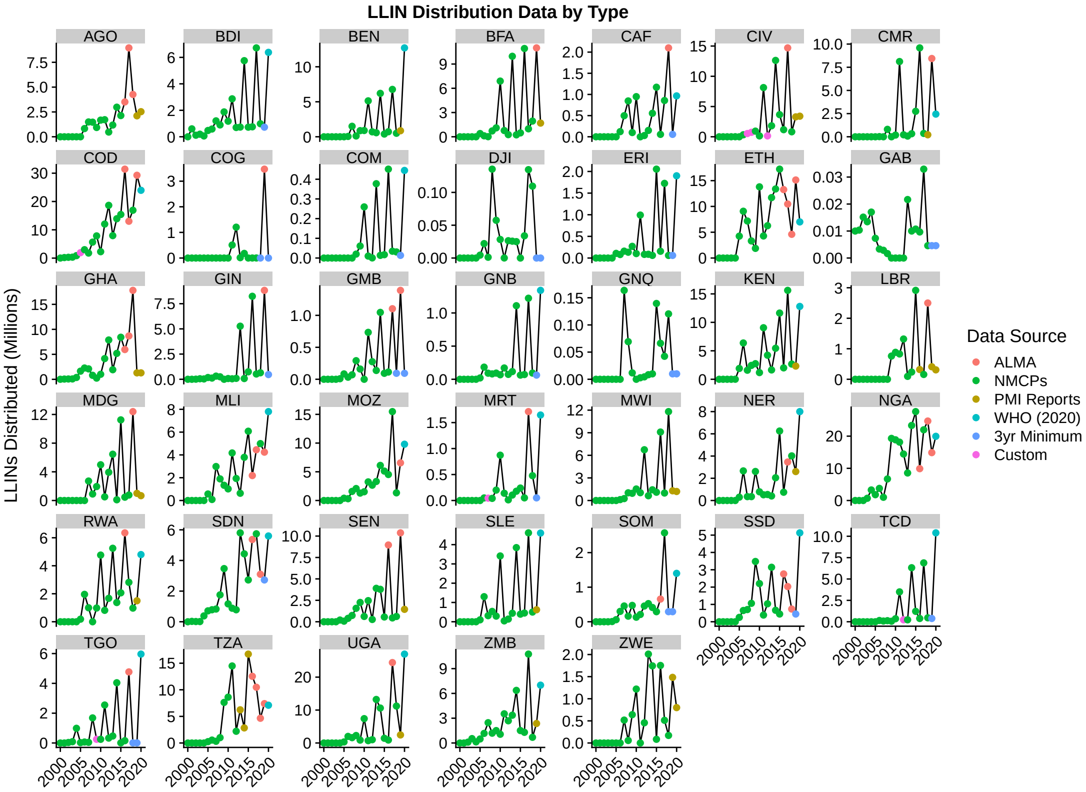

Supplementary Figure 1.1: Long-lasting insecticide treated net distribution by type, including direct data sources (red, green, gold, and cyan) and interpolated values (blue and pink).

1.3.3 Net Crop: Nationally-Representative Surveys

To obtain data on net crop, ITN ownership and household size indicators were collated and extracted from 161 nationally-representative household surveys conducted in Sub-Saharan Africa from 2000 to 2019. These included demographic health surveys (DHS), malaria indicator surveys (MIS), multiple indicator cluster surveys (MICS rounds 3, 4, and 5), AIDS indicator surveys (AIS), and one anemia and parasitemia survey (EA&P).

Data on the number of nets owned at the household level were available for 128 surveys in 36 countries (Supplementary Table 1.1, Supplementary Fig. 1.2). Of these, most reported net type for every net owned in a household, while some reported net type only on one net per household. For those surveys that reported net type for all nets in the household, the number of ITNs owned was determined by summing ITNs by type. For those surveys where data on net type was only available for one net per household, the overall survey-level proportion of nets by type (non-ITN, cITN, or LLIN) was determined and multiplied by the number of nets in the surveyed household to estimate the number of LLINs and cITNs owned by each household. These household-level values were aggregated using the appropriate survey weights to generate national-level estimates of mean cITNs per household, mean LLINs per household, and mean household size. The standard errors for each of these metrics was used to inform the width of survey-specific priors. Each survey was assigned to a single point in time, defined as the survey-weighted mean of the dates of household interviews.

For an additional 33 surveys in 20 countries, data were only available in aggregate from survey reports (Supplementary Table 3.1). For these, we collected measures of mean household size and mean cITNs and LLINs per household from the relevant report tables. The standard errors reported for each of these metrics was used to inform the width of survey-specific priors. Each survey was assigned to a single point in time, defined as the midpoint between the beginning and end of data collection for the survey.

1.3.4 Population

Population and population-at-risk (PAR) are from the central MAP database. Population estimates combine information from WorldPop, AfriPop, and IHME population databases. For more details see the supplementary materials of [7]. PAR was calculated by summing population in any pixel within the MAP-estimated

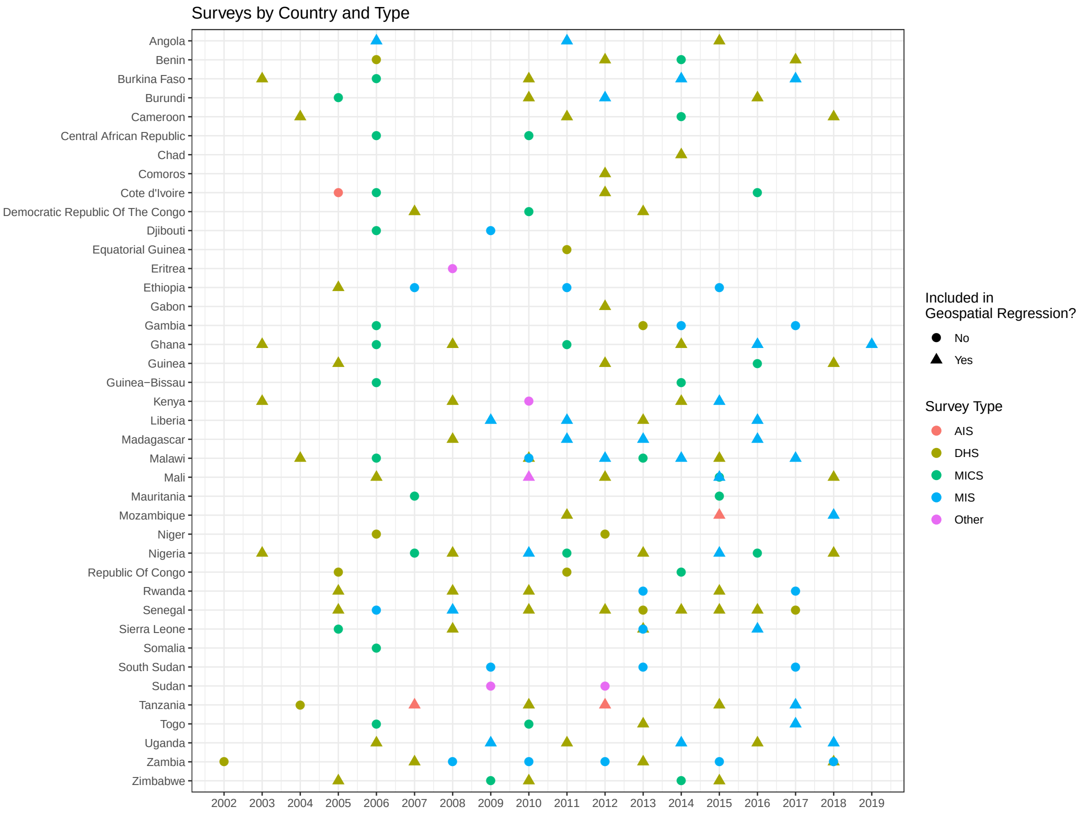

Supplementary Figure 1.2: Nationally-representative surveys included in this analysis, listed by country, year, and survey type.

Plasmodium falciparum transmission limits (downloadable at https://malariaatlas.org/explorer/#/).

Surveys used in ITN models.					
**Country**	**Survey Years**	**Source**	**No. of Clusters**	**No. of Individuals**	**Geospatial Data?**
Angola	2006-2007	MIS	115	13,952	Yes
Angola	2010-2011	MIS	238	39,951	Yes
Angola	2015-2016	DHS	625	72,870	Yes
Benin	2006	DHS	–	87,396	No
Benin	2011-2012	DHS	750	85,898	Yes
Benin	2014	MICS5	0	86,676	No
Benin	2017-2018	DHS	555	73,336	Yes
Burkina Faso	2003	DHS	400	58,844	Yes
Burkina Faso	2006	MICS3	–	37,070	No
Burkina Faso	2010	DHS	573	80,532	Yes
Burkina Faso	2014	MIS	252	38,393	Yes
Burkina Faso	2017-2018	MIS	245	36,307	Yes
Burundi	2005	MICS3	–	40,633	No
Burundi	2010-2011	DHS	376	40,983	Yes
Burundi	2012-2013	MIS	200	22,606	Yes
Burundi	2016-2017	DHS	554	76,528	Yes
Cameroon	2004	DHS	466	49,557	Yes
Cameroon	2011	DHS	578	70,294	Yes
Cameroon	2014	MICS5	–	51,031	No
Cameroon	2018-2019	DHS	430	58,474	Yes
Central African Republic	2006	MICS3	–	54,385	No
Central African Republic	2010	MICS4	–	52,355	No
Chad	2014-2015	DHS	624	96,005	Yes
Comoros	2012	DHS	252	23,580	Yes
Congo (Republic of)	2005	DHS	–	29,156	No
Congo (Republic of)	2011-2012	DHS	–	48,826	No
Congo (Republic of)	2014-2015	MICS5	–	67,159	No
Continued on next page					

Surveys used in ITN models, cont.					
**Country**	**Survey Years**	**Source**	**No. of Clusters**	**No. of Individuals**	**Geospatial Data?**
Cote d'Ivoire	2005	AIS	–	23,475	No
Cote d'Ivoire	2006	MICS3	–	54,402	No
Cote d'Ivoire	2011-2012	DHS	351	49,278	Yes
Cote d'Ivoire	2016	MICS5	–	65,109	No
Democratic Republic of the Congo	2007	DHS	300	46,496	Yes
Democratic Republic of the Congo	2010	MICS4	–	58,510	No
Democratic Republic of the Congo	2013-2014	DHS	536	93,147	Yes
Djibouti	2006	MICS3	–	28,781	No
Djibouti	2009	MIS	–	22,373	No
Equatorial Guinea	2011	DHS	–	19,745	No
Eritrea	2008	MIS	–	8,814	No
Ethiopia	2005	DHS	535	64,914	Yes
Ethiopia	2007	MIS	–	32,380	No
Ethiopia	2011	MIS	–	47,248	No
Ethiopia	2015	MIS	–	53,335	No
Gabon	2012	DHS	336	40,597	Yes
Gambia	2005-2006	MICS3	–	44,877	No
Gambia	2013	DHS	–	50,347	No
Gambia	2014	MIS	–	42,633	No
Gambia	2017	MIS	–	40,393	No
Ghana	2003	DHS	412	25,498	Yes
Ghana	2006	MICS3	–	24,947	No
Ghana	2008	DHS	411	45,297	Yes
Ghana	2011	MICS4	–	52,969	No
Ghana	2014	DHS	427	42,292	Yes
Ghana	2016	MIS	200	22,332	Yes
Ghana	2019	MIS	200	23,000	Yes
Continued on next page					

Surveys used in ITN models, cont.					
**Country**	**Survey Years**	**Source**	**No. of Clusters**	**No. of Individuals**	**Geospatial Data?**
Guinea	2005	DHS	295	36,950	Yes
Guinea	2012	DHS	300	43,876	Yes
Guinea	2016	MICS5	–	58,021	No
Guinea	2018	DHS	401	48,956	Yes
Guinea-Bissau	2006	MICS3	–	41,312	No
Guinea-Bissau	2014	MICS5	–	62,451	No
Kenya	2003	DHS	400	36,464	Yes
Kenya	2008-2009	DHS	398	37,790	Yes
Kenya	2010	MIS	–	26,946	No
Kenya	2014	DHS	1594	145,440	Yes
Kenya	2015	MIS	245	24,989	Yes
Liberia	2008-2009	MIS	150	21,876	Yes
Liberia	2011-2012	MIS	150	18,632	Yes
Liberia	2013	DHS	322	45,995	Yes
Liberia	2016	MIS	150	20,859	Yes
Madagascar	2008-2009	DHS	594	82,594	Yes
Madagascar	2011	MIS	267	39,337	Yes
Madagascar	2013	MIS	274	37,571	Yes
Madagascar	2016	MIS	358	47,640	Yes
Malawi	2004-2005	DHS	521	58,812	Yes
Malawi	2006	MICS3	–	131,021	No
Malawi	2010	DHS	849	115,027	Yes
Malawi		–	–	–	No
Malawi	2012	MIS	140	14,091	Yes
Malawi	2013-2014	MICS5	–	140,133	No
Malawi	2014	MIS	140	14,141	Yes
Malawi	2015-2016	DHS	850	117,833	Yes
Malawi	2017	MIS	150	16,330	Yes
Mali	2006	DHS	407	70,871	Yes
Mali	2010	EA & P	109	9,561	Yes
Mali	2012-2013	DHS	413	55,658	Yes
Mali	2015	MICS5	–	123,858	No
Mali	2015	MIS	177	38,705	Yes
Mali	2018	DHS	345	53,477	Yes
					Continued on next page

Surveys used in ITN models, cont.					
**Country**	**Survey Years**	**Source**	**No. of Clusters**	**No. of Individuals**	**Geospatial Data?**
Mauritania	2007	MICS3	–	59,572	No
Mauritania	2015	MICS5	–	69,675	No
Mozambique	2011	DHS	610	61,159	Yes
Mozambique	2015	AIS	306	32,051	Yes
Mozambique	2018	MIS	224	28,126	Yes
Niger	2006	DHS	–	46,403	No
Niger	2012	DHS	–	61,680	No
Nigeria	2003	DHS	362	34,609	Yes
Nigeria	2007	MICS3	–	124,840	No
Nigeria	2008	DHS	886	154,946	Yes
Nigeria	2010	MIS	239	30,134	Yes
Nigeria	2011	MICS4	–	143,686	No
Nigeria	2013	DHS	896	176,949	Yes
Nigeria	2015	MIS	326	37,772	Yes
Nigeria	2016-2017	MICS5	–	189,319	No
Nigeria	2018	DHS	1389	186,327	Yes
Rwanda	2005	DHS	462	46,715	Yes
Rwanda	2007-2008	DHS	249	31,741	Yes
Rwanda	2010-2011	DHS	492	55,280	Yes
Rwanda	2013	MIS	–	20,450	No
Rwanda	2014-2015	DHS	492	53,770	Yes
Rwanda	2017	MIS	–	19,901	No
Senegal	2005	DHS	376	66,506	Yes
Senegal	2006	MIS	–	28,918	No
Senegal	2008-2009	MIS	320	104,488	Yes
Senegal	2010-2011	DHS	391	74,941	Yes
Senegal	2012-2013	DHS	200	39,756	Yes
Senegal	2013	DHS	–	37,518	No
Senegal	2014	DHS	200	39,447	Yes
Senegal	2015	DHS	214	41,042	Yes
Senegal	2016	DHS	214	40,573	Yes
Senegal	2017	DHS	–	77,084	No
Sierra Leone	2005	MICS3	–	42,719	No
Sierra Leone	2008	DHS	353	40,426	Yes

Continued on next page

Surveys used in ITN models, cont.					
**Country**	**Survey Years**	**Source**	**No. of Clusters**	**No. of Individuals**	**Geospatial Data?**
Sierra Leone	2013	DHS	435	74,290	Yes
Sierra Leone	2013	MIS	–	36,395	No
Sierra Leone	2016	MIS	336	39,580	Yes
Somalia	2006	MICS3	–	33,959	No
South Sudan	2009	MIS	–	17,001	No
South Sudan	2013	MIS	–	18,337	No
South Sudan	2017	MIS	–	32,358	No
Sudan	2009	MIS	–	30,423	No
Sudan	2012	MIS	–	26,547	No
Tanzania	2004-2005	DHS	–	46,516	No
Tanzania	2007-2008	AIS	475	85,034	Yes
Tanzania	2009-2010	DHS	475	47,357	Yes
Tanzania	2011-2012	AIS	583	50,996	Yes
Tanzania	2015-2016	DHS	608	61,030	Yes
Tanzania	2017	MIS	442	45,814	Yes
Togo	2006	MICS3	–	30,542	No
Togo	2010	MICS4	–	29,573	No
Togo	2013-2014	DHS	330	45,432	Yes
Togo	2017	MIS	171	22,478	Yes
Uganda	2006	DHS	368	43,396	Yes
Uganda	2009-2010	MIS	170	20,916	Yes
Uganda	2011	DHS	404	102,538	Yes
Uganda	2014-2015	MIS	210	26,464	Yes
Uganda	2016	DHS	696	88,174	Yes
Uganda	2018-2019	MIS	340	44,489	Yes
Zambia	2001-2002	DHS	–	35,941	No
Zambia	2007	DHS	319	33,794	Yes
Zambia	2008	MIS	–	20,430	No
Zambia	2010	MIS	–	20,042	No
Zambia	2012	MIS	–	16,928	No
Zambia	2013-2014	DHS	721	78,486	Yes
Zambia	2015	MIS	–	16,129	No
Zambia	2018	MIS	–	18,384	No
Zambia	2018-2019	DHS	545	62,342	Yes

Continued on next page

Surveys used in ITN models, cont.					
**Country**	**Survey Years**	**Source**	**No. of Clusters**	**No. of Individuals**	**Geospatial Data?**
Zimbabwe	2005-2006	DHS	398	41,289	Yes
Zimbabwe	2009	MICS3	–	52,194	No
Zimbabwe	2010-2011	DHS	406	40,711	Yes
Zimbabwe	2014	MICS5	–	67,536	No
Zimbabwe	2015-2016	DHS	400	41,894	Yes

Supplementary Table 1.1: Surveys used in ITN models.

1.4 Model

1.4.1 Summary

National LLIN crop was estimated via a modified version of the mechanistic "stock and flow" model described below and in [2]. Briefly, a compartmental model was used to triangulate LLIN stock, distribution, and ownership data. Stock and distribution data were linked via a minimum function, mandating that no more than the available stock of nets could be distributed at a given time point. Distribution and ownership data were linked via a sigmoidal loss function to model discarding of LLINs over time. National cITN crop was modeled similarly, but no stock information was available for cITNs, so the upper bound on cITN distributions was omitted.

The model was run separately for each country. All parameters were fit separately by net type. After estimating LLIN and cITN crop, the two were summed into a single time series of national ITN crop. This was converted to ITN access via a household-size-based regression methodology described in [2] and below. All models were fitted in JAGS using the `rjags` package version 4.3.0, which produces draws from the posterior distributions of the fitted parameters. Each model generated 5,000 posterior draws, from which mean and 95% confidence intervals were computed to generate mean estimates and uncertainties for the conversion to access, 500 draws were randomly selected and propagated due to computational constraints.

For this analysis, the model described in [2] was refactored and rewritten in its entirety, but the model specification has remained largely constant. Key changes

are:

- Updating input data from net manufacturers, NMCPs, and surveys, including all information from ALMA and PMI;
- Changing from a time-varying to a static loss function parameterization to improve model identifiability;
- Placing a cap on excess stock distribution to ensure a reliable fit to survey data.

A formal model specification is presented below. To clarify notation, the process is described for a single country and for LLINs only. The specification for cITNs is identical except where noted.

1.4.2 Stock and Flow

All symbols and their definitions are shown in the tables below. Values drawn directly from data are represented by Roman characters, while parameters derived through model fitting are represented by Greek characters. Sigmas σ refer to parameter-specific standard errors, and hats ˆ over characters describe the modeled representations of data values.

Data on deliveries of LLINs to countries from manufacturers did not report uncertainty, but were assumed to be highly accurate. Therefore, we modelled LLIN deliveries m in year y as:

$$\sigma_y^m \sim \text{Unif}(0, 0.075)$$
$$\hat{m}_y \sim N(m_y, m_y\sigma_y^m)$$

This relationship allows for a small amount of noise in the number of LLIN deliveries. Similar data were not available for cITNs.

Data on ITN distributions also did not report uncertainty, but were assumed to be somewhat less accurate than manufacturer data given the presence of missing values and the difficulties inherent in collecting such information (see Supplement 1.3). Therefore, we placed modest priors on per-capita rates of ITN distribution:

Symbol	Meaning	Notes
$Y, y \in Y$	Year count, year index	
$Q, q \in Q$	Quarter count, quarter index	$Q = Y * 4 + 1$; see text for details
t	Continuous time	
$S, s \in S$	Survey count, survey index	
h	Household size	
P	National population	Source: MAP
m	Manufacturer deliveries of LLINs	Source: AMP
d	Reported cITN and LLIN distributions	Source: ALMA, NMCPS, and PMI
C, c^h, c^p	ITN crop (C), count per household c^h, and count per capita c^p	Source: Nationally representative surveys

Supplementary Table 1.2: Stock and flow parameter definitions: indices and data.

$$\sigma_y^d \sim \text{Unif}(0, 0.03)$$
$$\hat{d}_y \sim N(\frac{d_y}{P_y}, \sigma_y d)P_y$$

Since no net delivery data was available for cITNs, $\hat{d}_y^{cITN}$ was used without adjustment, while $\hat{d}_y^{LLIN}$ was further adjusted using $\hat{m}_y$.

An LLIN time series was constructed using information about both stock and distributions. Every year, LLINs were delivered to a country (acquisition of stock) and distributed (dispersal of stock). Not all acquired stock was necessarily dispersed each year, allowing for the possibility of stock accumulation over time. Stock at the beginning and end of every year was tracked using parameters α_y and ω_y.

In a given year, no more LLINs could be distributed than the available stock α_y, and no fewer could be distributed than $\hat{d}_y$. If more than $\hat{d}_y$ LLINs were available, a small portion of additional stock could be distributed to allow a closer fit to survey data. However, the model should not distribute a large amount of additional stock,

Symbol	Meaning
$\Gamma, \gamma^h, \gamma^p$	ITN crop (Γ), count per household γ^h, and count per capita γ^p
δ	Net distribution
α, ω	Initial and final LLIN stock for a given year
ϕ	Net distribution adjustment: proportion of surplus stock distributed
β	Shape parameter for Beta distribution of ϕ
κ, τ	Rate and duration parameters for net loss function

Supplementary Table 1.3: Stock and flow parameter definitions: modeled values.

both in the interest of hewing to data on net distributions and because countries may receive shipments of nets in anticipation of distributions in the following year. Therefore, the proportion of surplus stock to distribute, ϕ_y, was modeled as a Beta distribution with shape parameters 2 and β_y, truncated at 0.25. A narrow prior was placed on β such that the mean value of ϕ ranged between 0.07 and 0.09. The parameter ϕ can be thought of as a mechanistically-informed relaxation on the upper bound of the prior for LLIN distributions.

The LLIN distribution time series was constructed as follows. Beginning in year $y_0 = 2000$ and proceeding sequentially through each year:

$$\alpha_y = \hat{m}_{y_0} \text{ if } y = y_0, \quad \text{otherwise} \quad \alpha_y = \omega_{y-1} + \hat{m}_y$$
$$\delta_y = \min(\hat{m}_y, \hat{d}_y)$$
$$\beta_y \sim \text{Unif}(20, 24)$$
$$\phi_y \sim \text{Beta}(2, \beta_y) \quad \text{T}(0, 0.25)$$
$$\delta_y = \hat{d}_y + \phi_y(\alpha_y - \hat{d}_y)$$
$$\omega_y = \alpha_y - \delta_y$$

Where $T(\cdot)$ represents a truncated distribution. This yields an adjusted estimate for annual LLIN distributions, δ_y.

Next, annual ITN distributions δ_y were disaggregated the into quarterly net distributions δ_q. For each year y in Y:

1. Split the year into four quarters, $i \in 1:4$.
2. Define the proportion of ITNs distributed in each year-quarter, $p_{y,i} \sim \text{Unif}(0,1)$, raked such that $\sum_i p_{y,i} = 1$.
3. Define $\delta_{y,i} = \delta_y p_{y,i}$ for each quarter i.
4. Reindex such that quarters are indexed as δ_q rather than $\delta_{y,i}$.

The calculation was performed separately by net type, yielding quarterly estimates of net distribution δ_q^{LLIN} and δ_q^{cITN}. Since survey calibration requires interpolation between the beginning and end of each quarter, net distribution estimates were also calculated for the first quarter of year $Y+1$. These were assumed to be the same as distributions in the final quarter of the last year for which distribution data is available, $\delta_Q = \delta_{Q-1}$. From these quarterly distribution counts, net loss can be tracked.

Following [2], a smooth-compact loss function for nets was used, with:

$$\text{Loss}\ (t, \kappa, \tau) = e^{\kappa - \kappa/(1-(t/\tau)^2)}$$

if $t < \tau$, and $\text{Loss}(t, \kappa, \tau) = 0$ otherwise.

κ is therefore a rate parameter, while τ dictates the time by which all nets will be lost. Previous versions of the model constrained κ and allowed τ to vary widely. To further aid identifiability, here κ was fixed at a value of 20 and τ was given a wide prior $\tau \sim \text{Unif}(5.5, 20.7)$. After model fitting, net retention half-life was calculated analytically as the time t at which $\text{Loss}\ (t, \kappa, \tau) = 0.5$. To estimate net loss, for each net type a $Q \times Q$ matrix A was constructed, where each column j represents a quarter of net distribution and row i tracks net retention in subsequent quarters. Entries of the matrix were populated according to the following rules:

- $A_{ij} = 0$ if $i < j$ (nets have yet to be distributed)
- $A_{ij} = \delta_j$ if $i = j$ (nets were distributed in that quarter)
- $A_{ij} = \delta_j\ \text{Loss}(t, \kappa, \tau)$ if $i > j$, with $t = (i-j)\frac{1}{4}$.

Once A was populated, summing across columns generated estimated net crop by quarter, $\Gamma_q = \sum_{j \in 1:Q} A_{qj}$. Next, these quarterly distribution estimates were fit to the available survey data.

Each survey yielded an estimate of mean nets per household, c_s^h, and mean household size, h_s, each with standard errors. A national net crop C was estimated for each survey via $C_s = c_s^h \frac{P_y}{h_s}$, with P_y the national population in the year y in which survey s took place. Within the model, both nets per household and household size were assumed to be normally distributed, with means c_s^h and h_s and standard deviations from the standard errors of the survey data.

In the modeled time series, for each survey s conducted at time t estimated net crop was calculated as an interpolation between values at the beginning and end of the quarter q in which t falls:

$$\Gamma_t = (1-p)\Gamma_q + p\Gamma_{q+1}$$

where p represents the proportion of q that has elapsed by time t.

Net crop was then calibrated to surveys as:

$$C_s \sim N(\Gamma_t, \sigma_s^C) \quad \mathrm{T}(C_x - 3\sigma_s^C, C_s + 3\sigma_s^C)$$

Where the truncation constrains model estimates to hew closely to survey reports, which are highly reliable and widely used.

After calibration, estimates of net crop Γ_q^{LLIN} and Γ_q^{cITN} were summed to generate a time series of total ITN crop, Γ_q^{tot}. This is converted to per-capita ITNs using $\gamma_q^p = \frac{\Gamma_q^{tot}}{P_q}$. This is the main output of the stock and flow model, which is passed on to calculate household-size-specific indicators ρ_h^0 and μ_h to find a time series of ITN access by country.

1.4.3 Conversion to access

The simplest way to convert from ITN crop Γ to ITN access Λ^{acc} would be $\Lambda^{acc} = min(\frac{2\Gamma}{P}, 1)$, using the definition of access as ownership of two nets per person. However, this simple equation does not account for the overdispersion of nets to certain households, a well-documented phenomenon explored in depth in [8] and [2]. One of the most widely available predictors of sufficient access is household size, with nationally-representative surveys consistently showing that

Symbol	Meaning
ρ_h	Proportion of households of size h with no nets
μ_h	Mean net count given ownership of at least one net, among households of size h
Λ^{acc}	National-level ITN access

Supplementary Table 1.4: Parameter definitions for conversion of net crop to net access.

access to nets decreases with household size. Therefore, while converting from crop to access, country-specific household size distributions must be taken into account. As discussed in [2], the distribution of access among household sizes is well-represented by a zero-truncated Poisson distribution, requiring the estimation of both the proportion ρ_h of households with no nets and the mean net count μ_h among households with at least one net.

Following [2], this analysis defined a bivariate joint probability function $H = \mathbb{P}(h, n) \in \mathbb{R}^2$ as the probability mass of a household of size h owning n ITNs. For a given h, the conditional probability distribution $\mathbb{P}(n|h)$ was estimated as a zero-truncated Poisson distribution [9] characterized by ρ_h and μ_h. Therefore,

$$\mathbb{P}(n = 0|h) = p_h\rho_h$$
$$\mathbb{P}(n > 0|h) = p_h(1 - \rho_h)\frac{(\mu_h)^n}{n!(e^{\mu_h} - 1)}$$

with p_h the proportion of all households having size h. The distribution of household sizes p_h was calculated on a per-survey basis. H is a 10x40 matrix with rows representing household size h and columns representing net count n. These dimensions were chosen because some surveys only collect household size information up to ten people, and 40 is a sufficiently large net count to characterize all meaningful features of these distributions. The entries of H were renormalized as $H_{hn} = \frac{hH_{hn}}{\sum_{h,n} hH_{hn}}$ to convert them from probabilities among households to probabilities among the population. Using the definition of access as "the proportion of people able to sleep under an ITN, assuming one ITN per two people", national access among households of size h with n nets is then:

$$\Lambda^{acc}_{h,n} = \min(\frac{2n}{h}, 1)H_{hn}$$

And overall national access is the sum

$$\Lambda^{acc} = \sum_{h,n} \Lambda^{acc}_{h,n}.$$

Parameters ρ_h and μ_h were defined as polynomial functions of h and the national net crop per capita, γ^c:

$$\begin{aligned}
\beta_0 &\sim \text{Unif}(-50, 50)\\
\beta_1 &\sim \text{Unif}(-3, 3)\\
\beta_2 &\sim \text{Unif}(-1, 1)\\
\beta_3 &\sim \text{Unif}(-100, 100)\\
\beta_4 &\sim \text{Unif}(-300, 300)\\
\beta_5 &\sim \text{Unif}(-300, 300)\\
\tau &\sim \Gamma(0.1, 0.1)\\
\nu_h &= \beta_0 + \beta_1 h + \beta_2 h + \beta_3 \gamma^c + \beta_4 {\gamma_c}^2 + \beta_5 {\gamma_c}^3\\
\text{Empirical Logit}(\rho_h) &\sim N(\nu_h, \tau)
\end{aligned}$$

And:

$$\begin{aligned}
\beta_h &\sim \text{Unif}(-20, 20)\\
\tau &\sim \Gamma(0.1, 0.1)\\
\mu_h &\sim N(\beta_h, \tau)
\end{aligned}$$

See [2] for validation of these parameterizations. Equation coefficients were fit in Stan using `rstan` version 2.19.3 with all available household-level surveys. It should be noted that the mean of a zero-truncated Poisson with rate parameter λ is $\frac{\lambda}{(1-e^{-\lambda})}$ rather than simply λ. To coerce the mean of $\mathbb{P}$ to equal μ_h for all $n > 0$, the solution of $\mu_h = \frac{x}{(1-e^{-x})}$ was used rather than μ_h.

Quarterly values of ρ_h^0 and μ_h were predicted using these coefficients and the estimated net crop values γ^c from the stock and flow model, with uncertainty propagated at the draw level. These quarterly estimates were interpolated to the monthly time scale, and the monthly estimates used to calculate access as defined above. Thus, the final result of this modeling process was a monthly time series from 2000 through 2020 of ITN access at the national level for the 40 countries of interest.

1.5 Results: Crop-to-Access Regression and LLIN Median Retention Times

1.5.1 Crop-to-Access Regression Coefficients

Shown below are the outputs from the Stan regression to determine coefficients for the crop-to-access regression.

Variable	Mean (Lower 95%, Upper 95%)
β_0	4.07 (3.94, 4.19)
β_1	-0.286 (-0.327, -0.245)
β_2	0.0168 (0.0131, 0.0205)
β_3	-24.8 (-25.9, -23.6)
β_4	45.7 (41.2, 50.1)
β_5	-31.6 (-36.6, -26.6)
τ	0.406 (0.388, 0.425)

Supplementary Table 1.5: Regression coefficients for determining the proportion of households with no nets, ρ_h.

Household Size	Intercept	Slope (β_h)
1	0.15 (0.01, 0.3)	1.03 (0.5, 1.56)
2	0.24 (0.08, 0.39)	2.08 (1.54, 2.63)
3	0.41 (0.26, 0.56)	2.48 (1.94, 3.02)
4	0.52 (0.37, 0.67)	3.22 (2.68, 3.77)
5	0.65 (0.5, 0.8)	3.88 (3.35, 4.41)
6	0.76 (0.6, 0.91)	4.38 (3.82, 4.92)
7	0.81 (0.66, 0.96)	5.02 (4.49, 5.55)
8	0.83 (0.68, 0.98)	5.72 (5.18, 6.27)
9	0.87 (0.71, 1.02)	6.3 (5.76, 6.84)
10	1.06 (0.91, 1.21)	7.89 (7.35, 8.42)

Supplementary Table 1.6: Regression coefficients for determining mean nets per household, μ_h.

1.5.2 LLIN Median Retention Times

Shown below are the outputs from the full stock-and-flow model of median LLIN retention times by country.

LLIN Median Retention Times.			
**Country**	**ISO3**	**Median Retention Time in Years**	**Survey Count**
Angola	AGO	1.10 (1.01, 1.26)	3
Benin	BEN	1.07 (1.01, 1.17)	4
Burkina Faso	BFA	1.58 (1.41, 1.76)	5
Burundi	BDI	1.31 (1.14, 1.47)	4
Cameroon	CMR	3.49 (3.24, 3.78)	4
Central African Republic	CAF	1.90 (1.56, 2.26)	2
Chad	TCD	1.03 (1.01, 1.08)	1
Comoros	COM	2.13 (1.81, 2.39)	1
Congo (Republic of)	COG	2.91 (2.31, 3.65)	3
Cote d'Ivoire	CIV	1.69 (1.51, 1.86)	4
Democratic Republic of the Congo	COD	1.41 (1.15, 1.64)	3
Djibouti	DJI	1.05 (1.01, 1.13)	2
Equatorial Guinea	GNQ	3.59 (3.27, 3.79)	1
Eritrea	ERI	3.01 (1.94, 3.79)	1
Ethiopia	ETH	1.33 (1.19, 1.48)	4
Gabon	GAB	3.34 (2.63, 3.79)	1
Gambia	GMB	1.62 (1.39, 1.85)	4
Ghana	GHA	1.78 (1.67, 1.90)	7
Guinea	GIN	1.51 (1.28, 1.75)	4
Guinea-Bissau	GNB	1.38 (1.01, 2.16)	2
Kenya	KEN	2.26 (1.98, 2.58)	5
Liberia	LBR	1.03 (1.01, 1.07)	4
Madagascar	MDG	1.65 (1.48, 1.81)	4
Mali	MLI	2.81 (2.46, 3.14)	6
Mauritania	MRT	1.07 (1.01, 1.16)	2
Mozambique	MOZ	1.34 (1.21, 1.50)	3
Malawi	MWI	1.33 (1.21, 1.45)	9
Niger	NER	3.50 (3.25, 3.78)	2
Nigeria	NGA	2.22 (2.00, 2.47)	9
Rwanda	RWA	1.59 (1.48, 1.70)	6

Continued on next page

LLIN Median Retention Times, cont.			
**Country**	**ISO3**	**Median Retention Time in Years**	**Survey Count**
Senegal	SEN	1.35 (1.22, 1.48)	10
Sierra Leone	SLE	1.47 (1.31, 1.63)	5
Somalia	SOM	2.35 (1.02, 3.66)	1
South Sudan	SSD	1.02 (1.01, 1.04)	3
Sudan	SDN	2.91 (2.11, 3.77)	2
Tanzania	TZA	2.15 (1.88, 2.43)	6
Togo	TGO	2.42 (2.21, 2.61)	4
Uganda	UGA	1.66 (1.55, 1.78)	6
Zambia	ZMB	1.32 (1.21, 1.44)	9
Zimbabwe	ZWE	2.79 (2.26, 3.38)	5

Supplementary Table 1.7: Median retention times and 95% CI's for LLINs from the stock and flow model. Number of surveys used to fit each country model also listed.

1.6 Sensitivity Analysis

1.6.1 Methods

Each of the 40 countries included in the stock and flow model have between one and ten nationally-representative surveys available for use in model fitting (Supplementary Table 1.1). To assess sensitivity of this model framework to both survey count and individual survey values, we ran a series of tests on the eight countries with more than five survey points.

Sensitivity analyses included all available manufacturer and NMCP data, since the completeness of these data types does not change meaningfully across countries. Only sensitivity to survey data was tested.

Consider country c, with N_c available surveys. We reran the model $N_c - 1$ times, starting from a single survey point and adding an additional survey each round, to test the sensitivity of model estimates to the number of surveys included. To test the importance of the order of survey inclusion, we ran each sequence of $1 : Nc - 1$ models three different ways: in chronological order, in reverse chronological order, and in random order. The "random order" option shows only one possible permutation of non-chronological sequence, but it still gives some intuition into

**Country**	**Survey Count**
Ghana	6
Rwanda	6
Sierra Leone	6
Tanzania	6
Zambia	8
Malawi	9
Nigeria	9
Senegal	10

Supplementary Table 1.8: Countries included in sensitivity analysis.

the impact of survey inclusion order on model performance.

The main performance metric was out-of-sample LLIN crop root mean squared error (RMSE): that is, the RMSE of predicted LLIN crop vs observed LLIN crop for the surveys not included in each model run. We also compare estimates of LLIN median retention time between each held-out model and the full model.

1.6.2 Results

Supplementary Fig. 1.3 shows the out-of-sample RMSE (in units of millions of LLINs) for each country-iteration of the analysis. When surveys are added in chronological order, the RMSE trend is nonlinear, often peaking at some intermediate number of surveys before declining. Adding surveys in reverse chronological order shows a more stable trend, with RMSE generally declining with each additional survey. One major exception is Sierra Leone, which has a large peak in RMSE at three surveys. The random permutation approach splits the difference between these two, but generally behaves slightly more like the "reverse chronological" than the "chronological". For a given country-survey count, chronological RMSE is usually higher than reverse-chronological RMSE. Including only the low-net-count surveys early in the time series yields the highest RMSEs. Otherwise, there is not a consistent marker of which survey will have the highest impact on model fit. In Sierra Leone, for example, all models take a step change in RMSE upon the inclusion of the middle-of-the-series 2011 survey, while in Tanzania it is the two surveys at the end of the time series that most impact RMSE. For countries like Senegal, no one survey has an outsized impact.

Supplementary Fig. 1.4 shows the evolution of estimated LLIN median retention time (also called half-life). The solid line through each plot is the half-life estimated by the full model. Again here, with the exception of Sierra Leone, the "reverse chronological" sequence converges quickly to the value estimated by the full model, whereas the chronological sequence varies considerably before arriving at the full model's result. When just one survey is included, half-lives are more likely to be overestimates than underestimates of the full model value.

These results suggest that stock and flow time series fit to a small number of surveys, especially if those surveys occur early in the time series, may not be reliable estimators for LLIN half-life or net time series in the country they represent. As shown in Figure 5 of the main paper, countries with three or fewer surveys are likely to fall at the extreme ends of the LLIN half-life spectrum. In these countries, local knowledge and additional data collection will be crucial to understanding the true circumstances of net distribution, retention, and use.

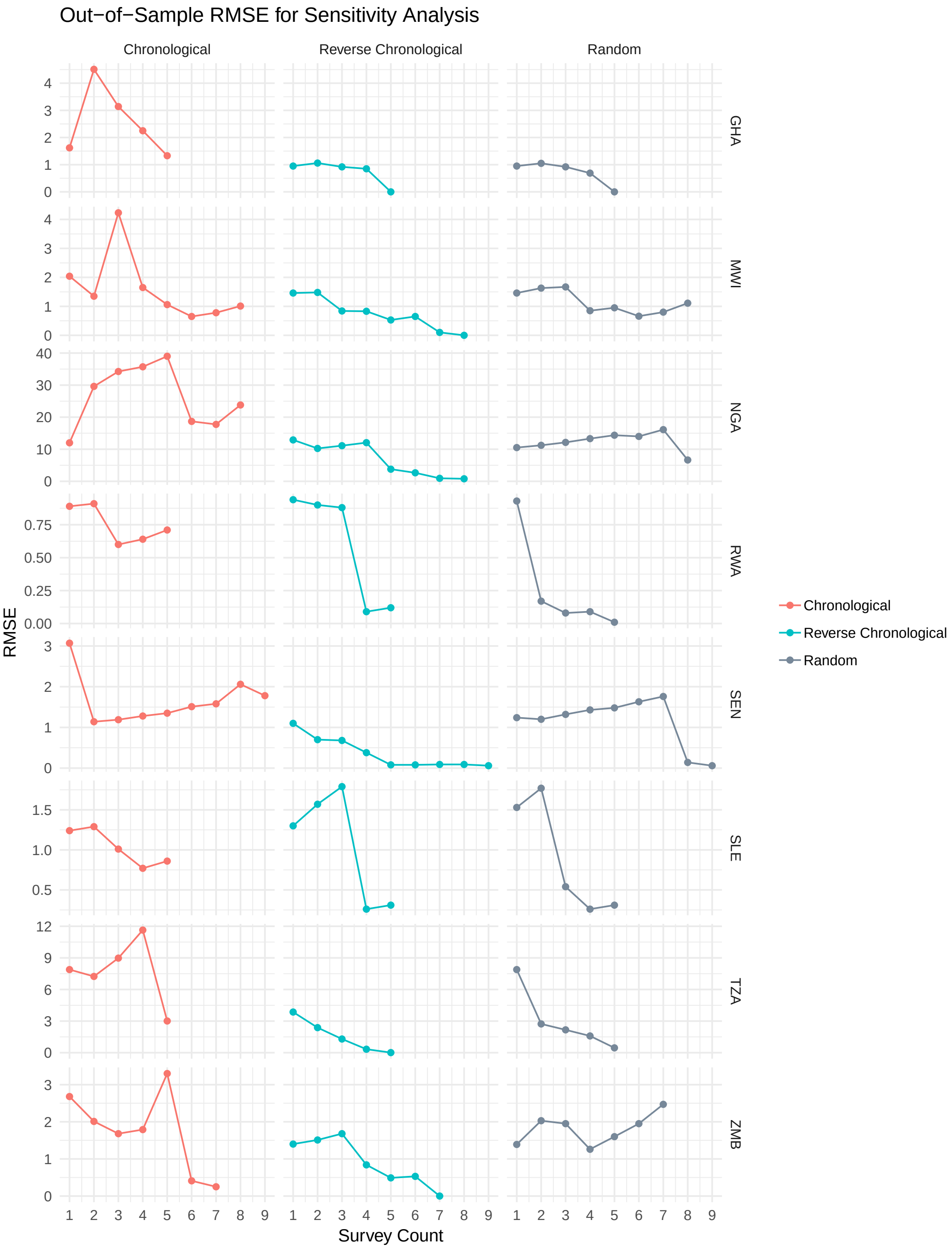

Supplementary Figure 1.3: Out-of-sample root mean squared error evolution in sensitivity analysis (Units: millions of LLINs)

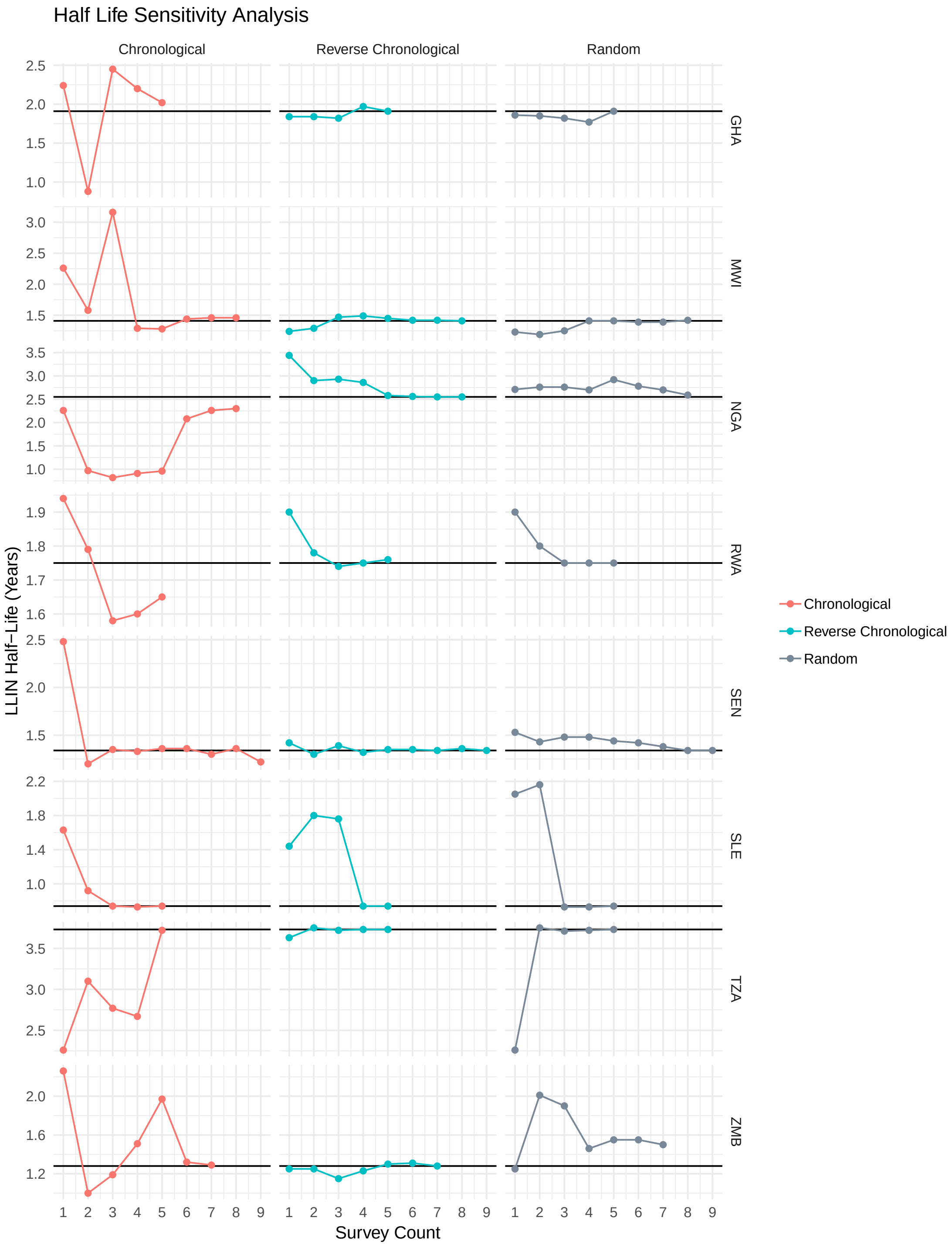

Supplementary Figure 1.4: Median retention time (or "half life") evolution in sensitivity analysis.

2 Supplementary Note 2: Spatiotemporal Regression Methods

A spatiotemporal regression framework was developed to derive 5km-by-5km pixel-level ITN access and use from the national-level access metrics estimated in the stock and flow model. First, national ITN access was disaggregated spatially, with survey data informing the difference between local and national access. Next, use was estimated from access, with survey data informing the gap between access and use over space and time. Additionally, a process similar to that for access was employed to estimate pixel-level nets-per-capita (NPC). NPC does not precisely capture ITN coverage, but does capture spatial heterogeneity in ITN distribution and retention that may be useful for crafting policy.

The present framing of this work is an adaptation of [10], with the following improvements:

- Cluster-level recalculation of national access to reflect local household size distributions;
- Addition of nets-per-capita as an outcome variable;
- Calculation of relative gain;
- Full propagation of uncertainty from stock and flow model;
- Addition of over two dozen new or previously-overlooked surveys;
- A fully refactored, publicly available codebase with Google Cloud functionality (https://github.com/bertozzivill/map-itn-cube/tree/publication-2021).

2.1 Definitions: Access Deviation, Use Gap, Nets-per-Capita Deviation

ITN "use" is the proportion of people who were reported to have slept under a net the night prior to the survey. ITN "nets-per-capita" is simply net crop divided by population. For all metrics, the population denominator was calculated from "de facto household size", defined as the number of people who slept in the household the night prior to the survey.

“Access deviation” is the difference between access in a specific location and the national mean access. Access deviation is positive when local access is greater than the national mean, and negative when local access is below the national mean. The “use gap” is the difference between access and use in any one location. The use gap is positive when not everyone with access to a net sleeps under it, negative when more than two people sleep under a single net, and zero when everyone who has access to a net sleeps under it respecting the “two people per net” guideline. Like access deviation, “NPC deviation” is the difference between nets-per-capita in a specific location within a country and the national mean nets-per-capita.

2.2 Data: National ITN Access, Household Survey Data, Covariates

2.2.1 National Net Access and Nets Per Capita

From the stock and flow model–see Supplement 1.4.

2.2.2 Household Survey Data

Ninety-five surveys from 28 countries with household-level data were used to fit the geospatial model. Indicators for (i) household size (defined as number of individuals that slept in the surveyed households the night prior to the survey), (ii) number of ITNs, and (iii) number of individuals that slept under an ITN the night prior to the survey were collected. The relevant cluster identifications were also collected to allow for aggregation to the cluster level. The final dataset contained 34,352 data points covering 28 countries and 17 years.

2.2.3 Covariates

To model the underlying mean function of the latent Gaussian model, we used a set of environmental and socioeconomic covariates consisting of rasterised satellite imagery across all of Africa at a 2.5 arc-minute (approximately 5km-by-5km) spatial resolution and monthly, annual, or static temporal resolution (Supplementary Table 2.1). All raster covariates were derived from high-resolution satellite images that were gap-filled [11] to eliminate missing data resulting primarily from

persistent cloud cover over equatorial forests. Some covariates are themselves modeled products that capture derived phenomena such as temperature suitability to mosquitoes or human accessibility to cities.

2.3 Latent Gaussian model

2.3.1 Cluster-level estimates of access, use, and nets per capita

Within a given household, the number of people with access to an ITN was calculated as $acc_h = min(2n_h, pop_h)$, with n_h the number of nets in that household. ITN access within a cluster was calculated as $\lambda^{acc} = \sum_h acc_h / \sum_h pop_h$. The underlying national access from the stock and flow model, Λ^{acc}, was adjusted at the cluster level to reflect variation in household size distribution, $\tilde{\Lambda}^{acc} = \sum_h pop_h \Lambda^{acc} / \sum_h pop_h$.

Cluster-level ITN use was calculated as $\lambda^{use} = \sum_c use_c / \sum_c pop_c$, with use_c the number of people who slept under an ITN the night prior to the survey in a given cluster. Cluster-level nets per capita was calculated as $\lambda^{npc} = \sum_c itn_c / \sum_n pop_c$, with itn_c the number of nets in a given cluster. Clusters were assigned to a time t based on a weighted average of sampling times. Values of λ^{acc}, λ^{use}, and λ^{npc} that fell within the same geographic pixel were aggregated to a single value.

2.3.2 Access Deviation, Use Gap, and Nets-Per-Capita Deviation

Due to the heterogeneity in net distribution volumes and times within countries and the sparsity of survey data, access is a highly nonstationary process which is challenging to predict at high resolution directly from λ^{acc} (Supplementary Fig. 2.1, top row). To create a stationary process that can readily be mapped, λ^{acc} was detrended by the underlying national mean. The resulting metric, access deviation, is therefore a measure of the subnational variation around an overall country mean (Supplementary Fig. 2.1, bottom row). Access deviation for a given pixel was calculated as $\lambda^{acc\_dev} = \lambda^{acc} - \tilde{\Lambda}^{acc}$. After model fitting and pixel-level prediction of λ^{dev}, pixel-level λ^{acc} was estimated by adding national mean access back onto access deviation. This process is identical for nets-per-capita deviation, λ^{npc_dev}.

Net use is similarly spatio-temporally non-stationary, motivating the estimation of the use gap instead. Use gap is simply the pixel-level difference between access and use, $\lambda^{gap} = \lambda^{acc} - \lambda^{use}$.

**Covariate**	**Source**	**Years Available**	**Resolution**
Aridity	WorldClim	-	Static
Elevation	SRTM	-	Static
Slope	SRTM	-	Static
NightTime Lights	VIIRS	2014	Static
Accessibility to Cities	MAP modeled output	2015	Static
Plasmodium falciparum Seasonality	MAP modeled output	-	Static
Potential Evotranspiration (PET)	WorldClim	-	Static
Topographic Moisture Index (TMI)	SRTM	-	Static
Population	MAP modeled output	2000-2020	Annual
Land Cover Class 2: Evergreen Broadleaf Forest	MODIS product	2001-2017	Annual
Land Cover Class 4: Deciduous Broadleaf Forest	MODIS product	2001-2017	Annual
Land Cover Class 5: Mixed Forest	MODIS product	2001-2017	Annual
Land Cover Class 6: Closed Shrublands	MODIS product	2001-2017	Annual
Land Cover Class 7: Open Shrublands	MODIS product	2001-2017	Annual
Land Cover Class 8: Woody Savannas	MODIS product	2001-2017	Annual
Land Cover Class 9: Savannas	MODIS product	2001-2017	Annual
Land Cover Class 10: Grasslands	MODIS product	2001-2017	Annual
Land Cover Class 11: Permanent Wetlands	MODIS product	2001-2017	Annual
Land Cover Class 12: Croplands	MODIS product	2001-2017	Annual
Land Cover Class 14: Cropland/Natural Vegetation Mosaic	MODIS product	2001-2017	Annual
Land Cover Class 16: Barren or Sparsely Populated	MODIS product	2001-2017	Annual
Land Cover Class 17: Water	MODIS product	2001-2017	Annual
Enhanced Vegetation Index (EVI)	MODIS product	2000-2017	Dynamic
Tassled Cap Wetness (TCW)	MODIS product	2000-2017	Dynamic
Daytime Land Surface Temperature (LST)	MODIS product	2000-2017	Dynamic
Nighttime Land Surface Temperature (LST)	MODIS product	2000-2017	Dynamic
Temperature Suitability Index (TSI)	MAP modeled output	2000-2017	Dynamic

Supplementary Table 2.1: Covariates used in all spatiotemporal models.

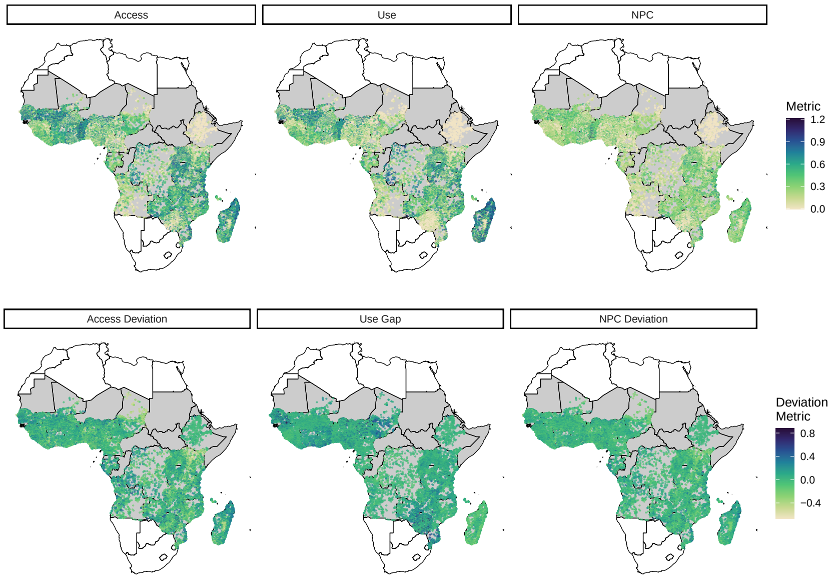

Supplementary Figure 2.1: Spatial stationarity of deviation metrics.

Both λ^{dev} and λ^{gap} were transformed via the empirical Logit to expand bounds to $[-\infty, \infty]$. This transformation is unnecessary for λ^{npc_dev}, which is not bounded between -1 and 1.

2.3.3 Latent Gaussian model for access deviation, use gap, and nets-per-capita deviation

All outcome metrics were fit using a Bayesian hierarchical model with a Gaussian likelihood and a Gaussian process prior:

$$\theta \sim \pi(\theta)$$
$$y|u,\theta \sim N(Au, \sigma_e^2)$$
$$u|\theta \sim N(\mu, Q^{-1})$$

with $\theta \in \{\sigma_e, \beta, \tau, \kappa, \phi\}$ a vector of hyperparameter priors; $y = \lambda^{acc\_dev}$, λ^{gap}, or λ^{npc_dev}; $\mu = \beta X$ a linear basis function for the fixed effects; σ_e^2 the variance for random Gaussian noise; u a Gaussian Markov Random Field (GMRF) representing

the spatiotemporal random effect; and A a sparse observation matrix that maps the GMRF to function evaluations at local observations.

The precision matrix Q on the GMRF was defined as a Kronecker product of a temporal process and a spatial process, $Q = Q_t \otimes Q_s$. Temporal correlation was modeled by first order autoregressive dynamics as $w_t = \phi w_{t-1}$ with $w_t \sim N(0, Q_t^{-1})$. The spatial process $w_s \sim N(0, Q_s^{-1})$ is the sparse finite element solution to the stochastic partial differential equation

$$(\kappa^2 - \Delta)^{\alpha/2} \tau u(s) = W(s)$$

where Δ is the Laplacian operator, κ is the spatial scale parameter, α is the spatial smoothness parameter (fixed at $\alpha = 2$), τ controls the variance, and $W(s)$ is the spatial white noise process. Because the spatial process covers a substantial portion of the Earth's surface, s was defined on a spherical manifold in Cartesian $\mathbb{R}^3$. For more details see [12].

The models were fit in R using the Integrated Nested Laplace Approximation (INLA version 20.03.17, `https://www.r-inla.org/`). To allow for correct posterior coverage and credible intervals under the Laplace approximation we transformed both access and use via the inverse hyperbolic sine function, $arsinh(x) = ln(x + \sqrt{x^2 + 1})$, thereby allowing the response in the model to be as close to Gaussian as possible. For a given scaling parameter ρ and response $x \in \{\lambda^{acc\_dev}, \lambda^{gap}, \lambda^{npc\_dev}\}$, with n observations, we defined $ihs(x, \rho) = (arsinh(x)\rho)/\rho$. The optimal ρ was found by minimizing the following log-likelihood:

$$l(\rho, x) \propto -nlog(\sum (x - \bar{x})^2) - \sum log(1 + \rho^2 x^2).$$

For more details see [13].

2.4 Results

2.4.1 National Time Series of Regression Predictions

Plotted here are national-level time series of the three regression outputs. On the national level, by definition, both access deviation (Supplementary Fig. 2.2) and NPC deviation (Supplementary Fig. 2.3) should be close to zero. However,

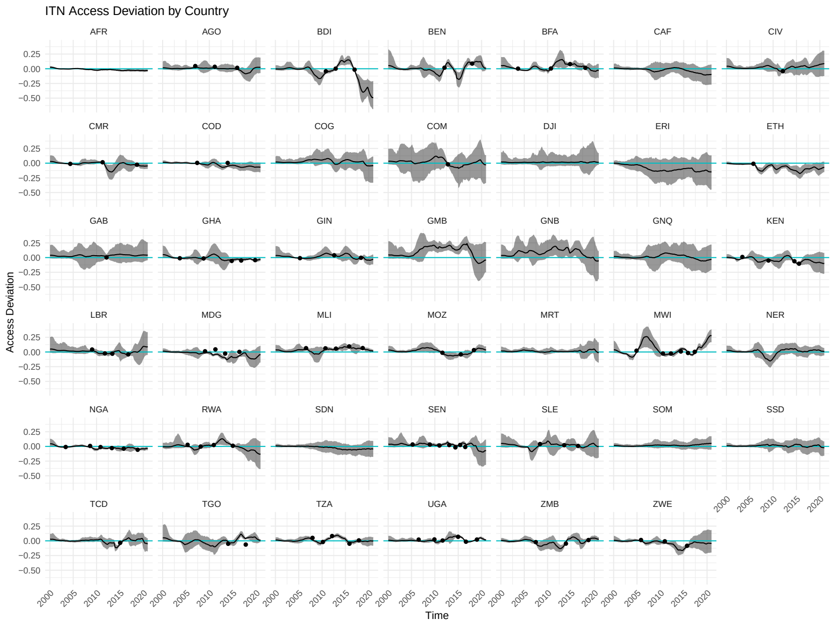

Supplementary Figure 2.2: ITN Access Deviation by Country. Solid curves show mean values, and shaded areas represent 95% confidence intervals. Survey data mean values shown as solid circles. AFR: Africa. All other abbreviations are ISO3 codes.

because national mean estimates (blue horizontal lines) are derived from the stock and flow model for these two measures, the survey data can show nonzero values in country-years where the stock and flow model did not perfectly fit the surveys, and the regression estimates will be similarly nonzero.

Use gap values are not constrained to be near zero. Supplementary Fig. 2.4 also demonstrates the stronger seasonal patterns detected in the use gap regression. While use rate is a derived output that was not directly fitted to data, we include time series of it here for reference (Supplementary Fig. 2.5).

2.4.2 Access vs NPC: Model and Data

Figure 4 demonstrates the curvature of the relationship between ITN access and NPC for modeled country-months in the year 2020. Supplementary Fig. 2.6 shows the consistency of this relationship by plotting survey data on the cluster level along with modeled estimates on the country-month level for all years in which survey data was available.

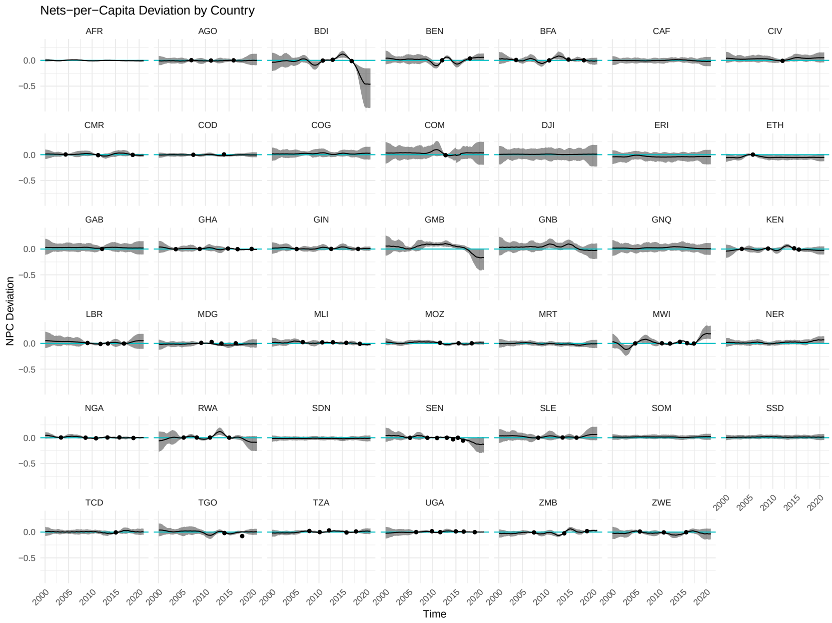

Supplementary Figure 2.3: Nets-per-Capita Deviation by Country. Solid curves show mean values, and shaded areas represent 95% confidence intervals. Survey data mean values shown as solid circles. AFR: Africa. All other abbreviations are ISO3 codes, see Supplementary Table 1.7 for a key to country names. Solid blue line at zero included as a reference point.

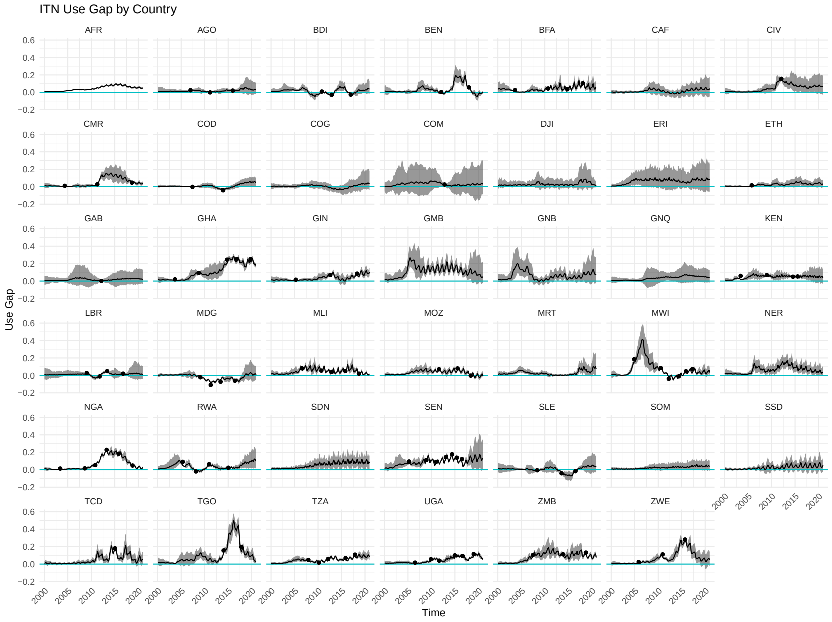

Supplementary Figure 2.4: ITN Use Gap by Country. Solid curves show mean values, and shaded areas represent 95% confidence intervals. Survey data mean values shown as solid circles. AFR: Africa. All other abbreviations are ISO3 codes, see Supplementary Table 1.7 for a key to country names. Solid blue line at zero included as a reference point.

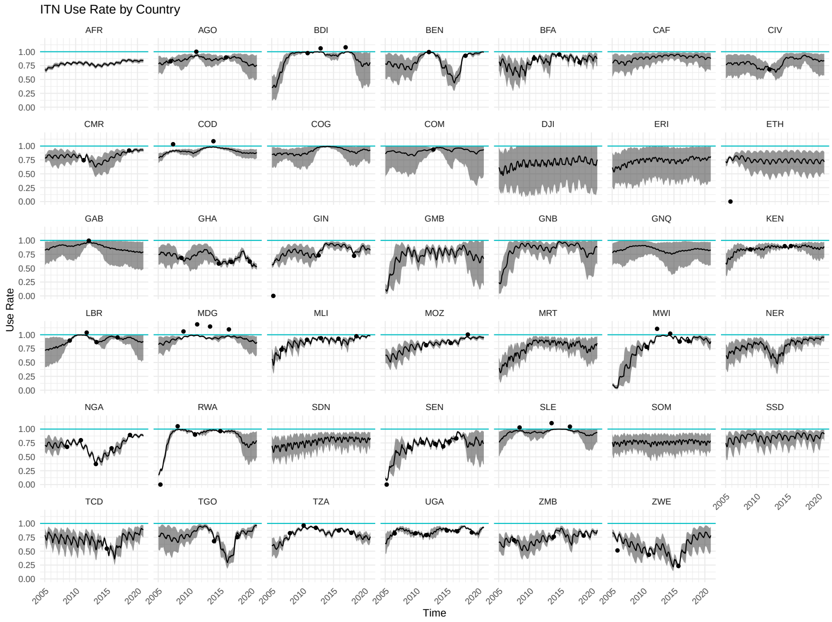

Supplementary Figure 2.5: ITN Use Rate by Country. Solid curves show mean values, and shaded areas represent 95% confidence intervals. Survey data mean values shown as solid circles. AFR: Africa. All other abbreviations are ISO3 codes, see Supplementary Table 1.7 for a key to country names. Solid blue line at zero included as a reference point.

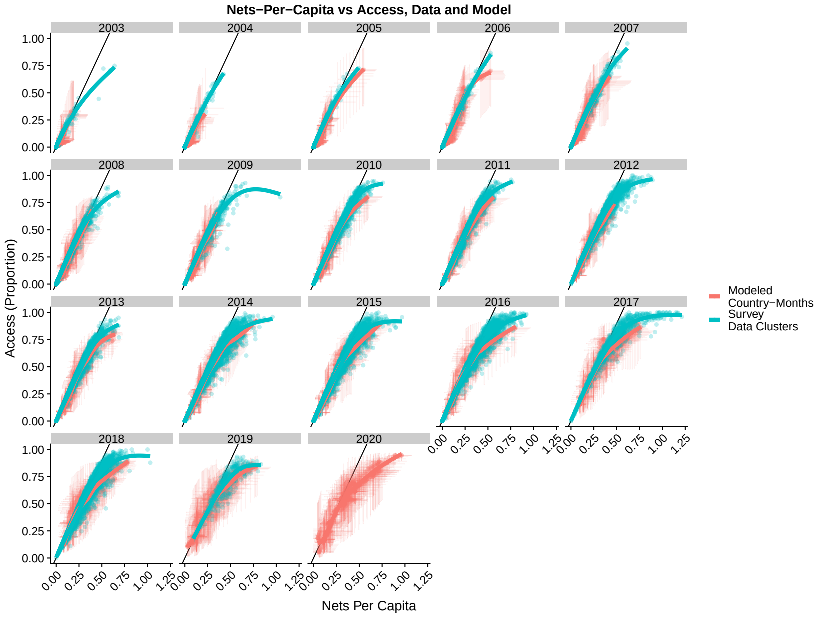

Supplementary Figure 2.6: Comparison of modeled (red) and raw data (blue) relationship between nets-per-capita and ITN access.

2.4.3 Assessing the Impact of Imperfect Allocation: Optimal vs True Access

Another way to assess the impact of less-than-perfect net allocation is to calculate a counterfactual "optimal access" metric based on the number of nets-per-capita, $\min(2 * \mathrm{NPC}, 1)$. Plotting this value against estimates for access (Supplementary Fig. 2.7) gives an upper bound of what ITN access could look like with improved allocation.

2.4.4 Example of Exceedance Surfaces for Uncertainty Visualization

The main manuscript communicates geospatial uncertainty using an uncertainty quantile approach. Another way of visualizing this uncertainty is through an exceedance approach. For Bayesian models such as these, exceedance surfaces demonstrate the proportion of posterior draws that are above or below a given threshold. As an example, Supplementary Fig. 2.8 shows posterior mean maps (central column) along with several positive and negative exceedance surfaces. The top row demonstrates that all 200 posterior draws showed an access estimate below 0.3 in Angola in 2020, whereas in Botswana only a fraction of draws were

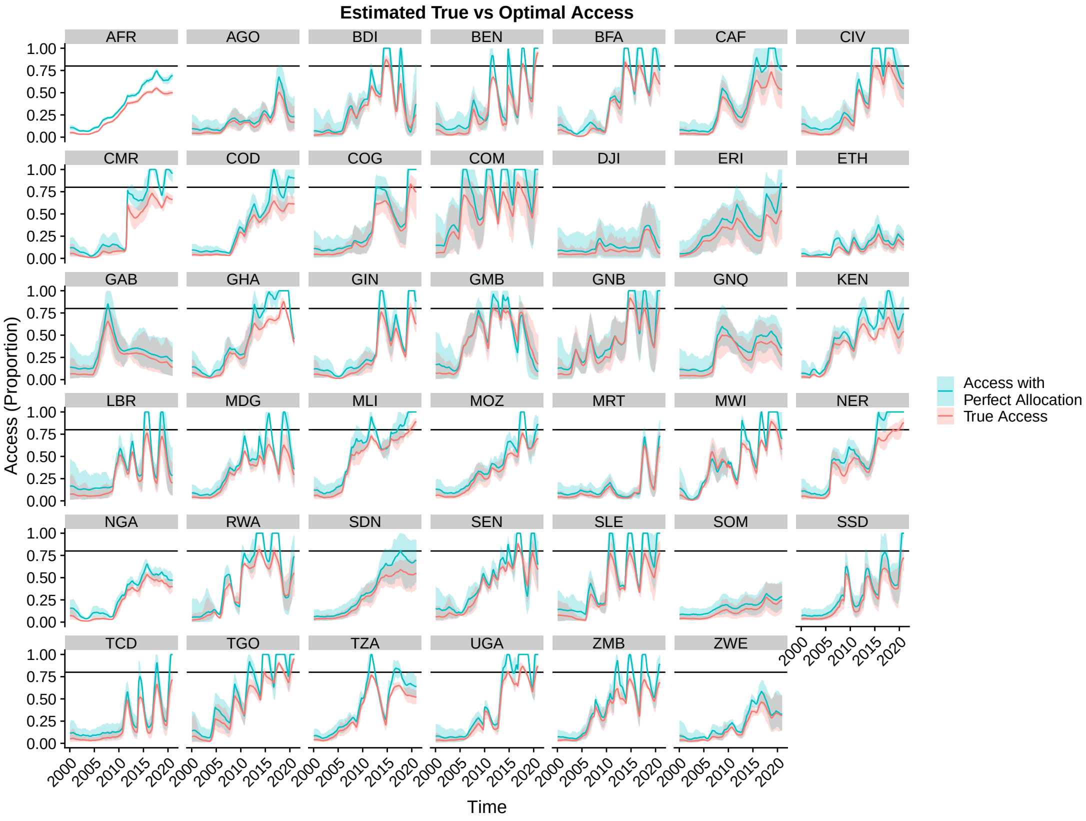

Supplementary Figure 2.7: ITN Estimated True vs Optimal Access by Country. Estimated true access shown in red, and hypothetical access with perfect allocation shown in blue. Solid curves show mean values, and shaded areas represent 95% confidence intervals.

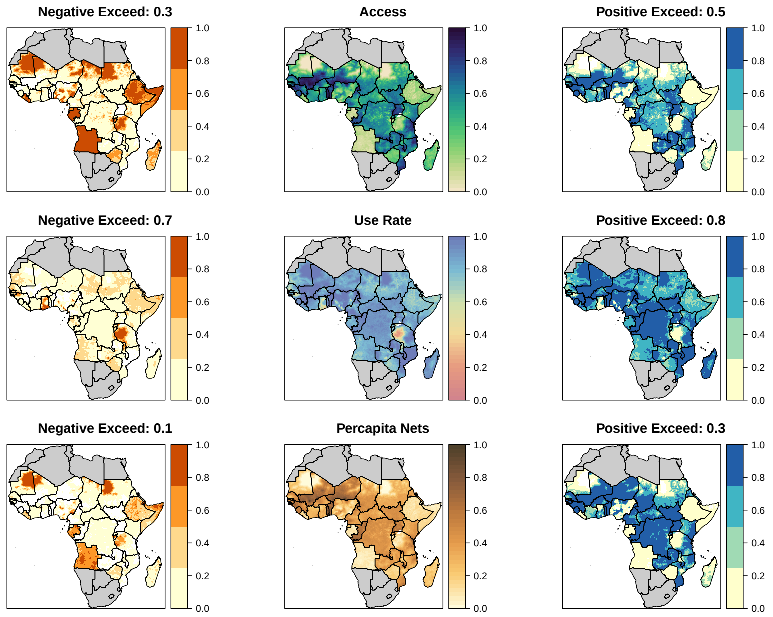

Supplementary Figure 2.8: Example 2020 exceedance surfaces.

below this threshold. In contrast, almost all of Botswana had posterior draws below 0.5 in this same year. An interactive visualization to explore a range of cutoffs is available at the Malaria Atlas Project website at `https://malariaatlas.org/research-project/metrics-of-insecticide-treated-nets-distribution`.

2.4.5 Regression Coefficients and Model Validation

Supplementary Fig. 2.9 shows coefficient values for all model covariates. Note that these values are in transformed variable space, not level space.

Supplementary Fig. 2.10 plots true vs predicted values for all survey data points. Posterior coverage was evaluated by leave-one-out probability integral transforms (PIT). For well-calibrated posteriors, the distribution of PITs should be close to Uniform. PIT distributions showed acceptable posterior coverage for all models, though the NPC regression will require more adjustment in future iterations to ensure proper calibration (Supplementary Fig. 2.11).

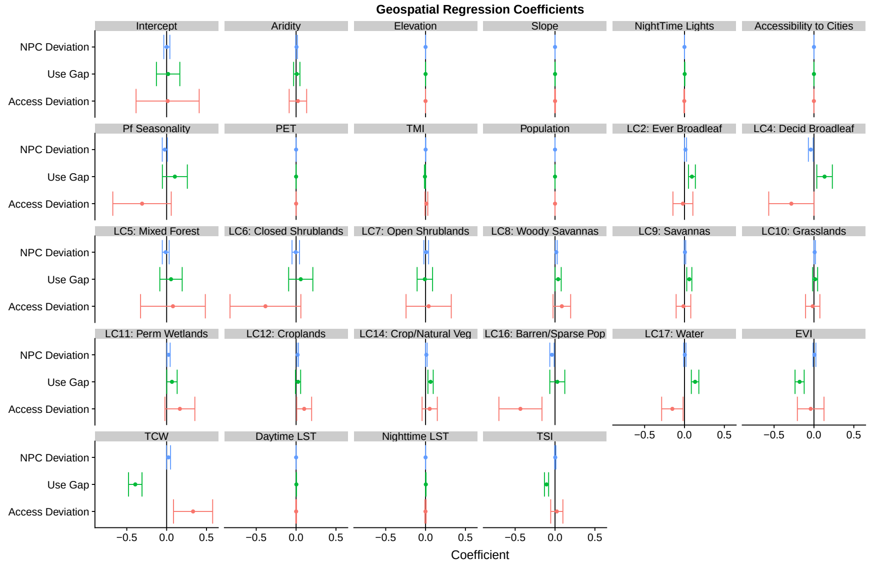

Supplementary Figure 2.9: Geospatial Regression Coefficients. Dots represent mean values, and error bars represent 95% uncertainty intervals. For abbreviation definitions see Supplementary Table 2.1.

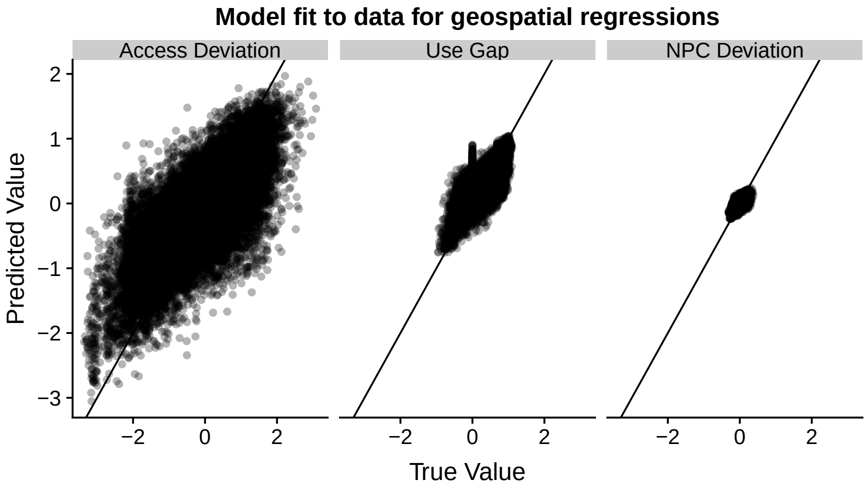

Supplementary Figure 2.10: Geospatial regression model fits.

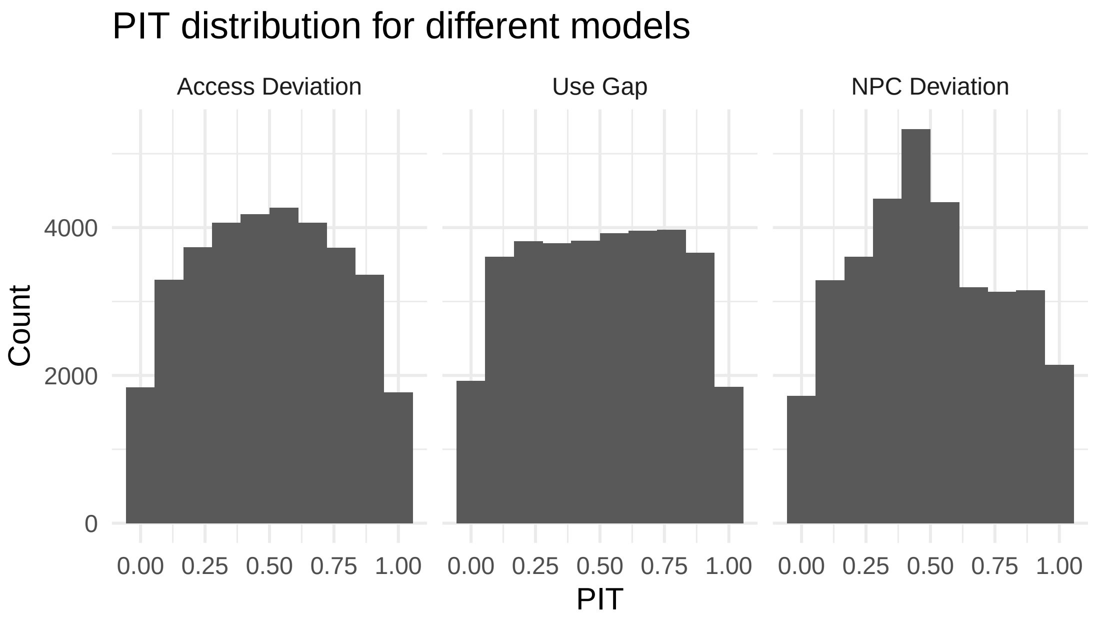

Supplementary Figure 2.11: Geospatial regression PIT distributions.

2.5 Covariate Selection

2.5.1 Methods

The full set of covariates (Supplementary Table 2.1) was selected during a previous iteration of this analysis [10]. The temperature- and aridity- based covariates were selected on the basis of a documented relationship between these variables and net use [14, 15]. The remaining variables were selected either as proxies for socioeconomic status (nighttime lights, population, access to cities) or as proxies for other spatially-varying cultural behaviors that may impact net use (land cover types). Because the outputs of this analysis are in turn used as inputs into a regression model to estimate malaria prevalence [7, 16], covariate selection was also constrained to not overlap with the covariates used in the downstream model to avoid circularity.

Three covariate combinations were assessed for predictive performance in the access deviation and use gap models: The full set of available covariates, the full set excluding landcover covariates, and a custom set based on the magnitude and significance of effects in the full model (Supplementary Table 2.2). Models were compared on the basis of pointwise leave-one-out predictive density $loo_i =$

log $p(y_i|y_{-i})$ and pointwise Watanabe-Akaike Information Criterion (WAIC).

2.5.2 Results

The model utilizing the full set of covariates performed best by all metrics except for WAIC in the access deviation regression, for which it performed almost identically to the "No Landcover" model (Supplementary Table 2.3). Based on these results, the full covariate set was utilized in the final model.

2.6 Uncertainty Propagation

Both JAGS and INLA return samples from the posterior distributions of the parameters of interest, allowing summary statistics to be explicitly computed as the mean and 95% percentile ranges across these samples. The ideal way to propagate uncertainty would be to run a separate INLA regression for each of the N_{sf} stock-and-flow posterior samples, but this was beyond our computational capacity. Instead, the mean value across the $N_{sf} = 500$ posterior INLA samples was used as the "national mean" from which access deviation and NPC deviation were computed. Then, $N_{geo} = 200$ posterior samples were drawn from each geospatial regression (access deviation, use gap, and NPC deviation). To compute access, use, and NPC at the draw level, 200 samples were selected at random from the stock-and-flow draws and these were added to the INLA samples in place of the stock-and-flow mean values to recapture some stock-and-flow uncertainty in the final result.

2.7 Relative Gain

By separately modeling access and use, this analysis was able to distinguish whether lack of net access or lack of net use was the primary driver of low net coverage for those areas that do not achieve universal coverage.

This analysis required the answers to two questions. First, how high could ITN use be if access were kept constant but the use rate were maximized? Second, how high could ITN use be if the use rate were kept constant but access were maximized? These two questions correspond to maps of ITN access and the ITN use rate (use/access) in a given year, respectively. By subtracting true use from

**Covariate**	**Resolution**	**Included in "No Land-cover"**	**Included in Custom Set**
Aridity	Static	Yes	Yes
Elevation	Static	Yes	No
Slope	Static	Yes	No
NightTime Lights	Static	Yes	No
Accessibility to Cities	Static	Yes	No
Plasmodium falciparum Seasonality	Static	Yes	Yes
Potential Evotranspiration (PET)	Static	Yes	No
Topographic Moisture Index (TMI)	Static	Yes	No
Population	Annual	Yes	No
Land Cover Class 2: Evergreen Broadleaf Forest	Annual	No	Yes
Land Cover Class 4: Deciduous Broadleaf Forest	Annual	No	Yes
Land Cover Class 5: Mixed Forest	Annual	No	No
Land Cover Class 6: Closed Shrublands	Annual	No	No
Land Cover Class 7: Open Shrublands	Annual	No	No
Land Cover Class 8: Woody Savannas	Annual	No	No
Land Cover Class 9: Savannas	Annual	No	Yes
Land Cover Class 10: Grasslands	Annual	No	Access Deviation Only
Land Cover Class 11: Permanent Wetlands	Annual	No	Yes
Land Cover Class 12: Croplands	Annual	No	Use Gap Only
Land Cover Class 14: Cropland/Natural Vegetation Mosaic	Annual	No	No
Land Cover Class 16: Barren or Sparsely Populated	Annual	No	Access Deviation Only
Land Cover Class 17: Water	Annual	No	Yes
Enhanced Vegetation Index (EVI)	Dynamic	Yes	Yes
Tassled Cap Wetness (TCW)	Dynamic	Yes	Yes
Daytime Land Surface Temperature (LST)	Dynamic	Yes	No
Nighttime Land Surface Temperature (LST)	Dynamic	Yes	No
Temperature Suitability Index (TSI)	Dynamic	Yes	Yes

Supplementary Table 2.2: Covariates used in covariate selection tests.

Model	Covariate Type	LOO	WAIC
**Access Deviation**	Full Model	**-49,951**	91,705
	No Landcover	-45,951	**91,670**
	Custom	-46,130	92,048
**Use Gap**	Full Model	**903.6**	**-1902.7**
	No Landcover	888.8	-1872.6
	Custom	859.6	-1814.3

Supplementary Table 2.3: Validation metrics for covariate sets.

each surface, the percentage point gain in coverage from each of these scenarios was calculated. The relative gain of one scenario over another was then assessed by comparing the two gain surfaces.

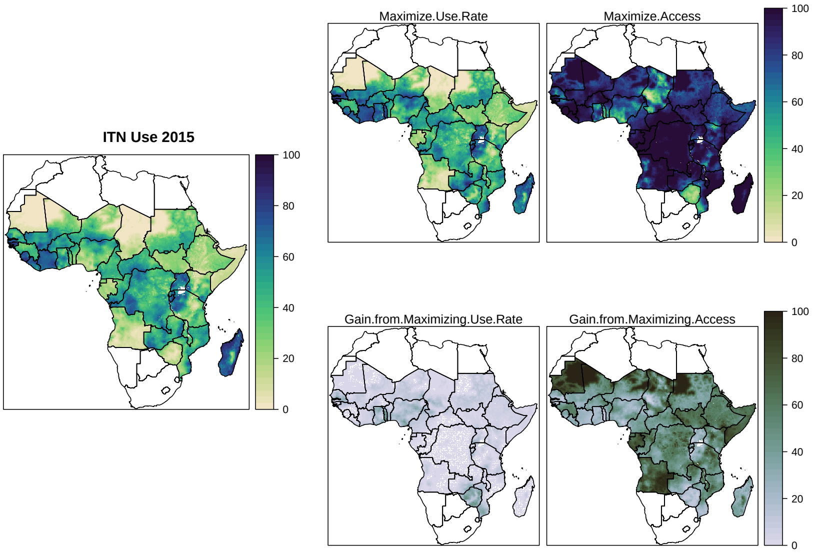

Supplementary Figure 2.12: Relative gain example from 2015. The leftmost plot shows estimated use, the top two plots on the right show use if the use rate were maximized versus if access were maximized, and the bottom two plots on the right show the percentage point increase in use for these two scenarios.

3 Supplementary Note 3: Additional Plots and Tables

3.1 Sources for Report-Only Surveys

Some surveys were not available at the household level, so summary measures were extracted from survey reports. This section details those surveys and the locations and page numbers in which the data was extracted.

Report-only survey details.

Country	ISO3	Survey Years	Source	Report Page	Link
Burkina Faso	BFA	2006	MICS3	6	https://mics-surveys-prod.s3.amazonaws.com/MICS3/West%20and%20Central%20Africa/Burkina%20Faso/2006/Final/Burkina%20Faso%202006%20MICS_French.pdf
Burundi	BDI	2005	MICS3	24	https://mics-surveys-prod.s3.amazonaws.com/MICS3/Eastern%20and%20Southern%20Africa/Burundi/2005/Final/Burundi%202005%20MICS_French.pdf

Continued on next page

Report-only survey details, cont.					
**Country**	**ISO3**	**Survey Years**	**Source**	**Report Page**	**Link**
Central African Republic	CAR	2006	MICS3	30	https://mics-surveys-prod.s3.amazonaws.com/MICS3/West%20and%20Central%20Africa/Central%20African%20Republic/2006/Final/Central%20African%20Republic%202006%20MICS_French.pdf
Code d'Ivoire	CIV	2006	MICS3	26	https://mics-surveys-prod.s3.amazonaws.com/MICS3/West%20and%20Central%20Africa/C%C3%B4te%20d%27Ivoire/2006/Final/Cote%20d%27Ivoire%202006%20MICS_French.pdf
Djibouti	DJI	2006	MICS3	25	https://mics-surveys-prod.s3.amazonaws.com/MICS3/Middle%20East%20and%20North%20Africa/Djibouti/2006/Final/Djibouti%202006%20MICS_French.pdf

Continued on next page

Report-only survey details, cont.					
**Country**	**ISO3**	**Survey Years**	**Source**	**Report Page**	**Link**
Djibouti	DJI	2009	MIS	15	https://malariaatlas.org/media/PDF/Djibouti_MIS_Report.pdf
Ethiopia	ETH	2007	MIS	10	https://www.cartercenter.org/resources/pdfs/news/health_publications/malaria/Ethiopia_2007_MIS_Report.pdf
Ethiopia	ETH	2011	MIS	12	https://www.ephi.gov.et/images/downloads/ethiopia-mis2011-report.pdf
Ethiopia	ETH	2015	MIS	20	https://www.malariasurveys.org/documents/Ethiopia_MIS_2015.pdf
Equatorial Guinea	GNQ	2011	DHS	17	https://dhsprogram.com/pubs/pdf/FR271/FR271.pdf
Gambia	GMB	2005-6	MICS3	15	https://mics-surveys-prod.s3.amazonaws.com/MICS3/West%20and%20Central%20Africa/Gambia/2005-2006/Final/Gambia%202005-06%20MICS_English.pdf
Continued on next page					

Report-only survey details, cont.					
**Country**	**ISO3**	**Survey Years**	**Source**	**Report Page**	**Link**
Gambia	GMB	2014	MIS	18	http://ccmgambia.org/images/pdfs/Final-Report-MIS-2014.pdf
Gambia	GMB	2017	MIS	13	https://www.malariasurveys.org/documents/GMIS%202017%20final%20report.pdf
Ghana	GHA	2006	MICS3	8	https://mics-surveys-prod.s3.amazonaws.com/MICS3/West%20and%20Central%20Africa/Ghana/2006/Final/Ghana%202006%20MICS_English.pdf
Guinea-Bissau	GNB	2006	MICS3	30	https://mics-surveys-prod.s3.amazonaws.com/MICS3/West%20and%20Central%20Africa/Guinea-Bissau/2006/Final/Guinea-Bissau%202006%20MICS_French.pdf

Continued on next page

Report-only survey details, cont.					
**Country**	**ISO3**	**Survey Years**	**Source**	**Report Page**	**Link**
Malawi	MWI	2006	MICS3	10	https://mics-surveys-prod.s3.amazonaws.com/MICS3/Eastern%20and%20Southern%20Africa/Malawi/2006/Final/Malawi%202006%20MICS_English.pdf
Malawi	MWI	2010	MIS	13	https://files.givewell.org/files/DWDA%202009/AMF/Malawi_MIS_2010_Final.pdf
Mauritania	MRT	2007	MICS3	10	http://mics.unicef.org/surveys
Nigeria	NGA	2007	MICS3	6	https://mics-surveys-prod.s3.amazonaws.com/MICS3/West%20and%20Central%20Africa/Nigeria/2007/Final/Nigeria%202007%20MICS_English.pdf
Senegal	SEN	2013	DHS	12	https://dhsprogram.com/pubs/pdf/FR288/FR288eng.pdf
Sierra Leone	SLE	2005	MICS3	7	https://mics.unicef.org/surveys
Sierra Leone	SLE	2013	MIS	18	https://dhsprogram.com/pubs/pdf/MIS15/MIS15.pdf
					Continued on next page

Report-only survey details, cont.					
**Country**	**ISO3**	**Survey Years**	**Source**	**Report Page**	**Link**
Somalia	SOM	2006	MICS3	23	https://mics-surveys-prod.s3.amazonaws.com/MICS3/Eastern%20and%20Southern%20Africa/Somalia/2006/Final/Somalia%202006%20MICS_English.pdf
South Sudan	SSD	2009	MIS	9	https://www.malariasurveys.org/documents/SS%202009_Malaria%20Indicator%20Survey%20Report.pdf
South Sudan	SSD	2013	MIS	8	https://www.malariasurveys.org/documents/MIS%20Report%202013%20Final%20Pages.pdf
South Sudan	SSD	2017	MIS		https://www.malariasurveys.org/documents/SSMIS%202017%20final%20report%202019%2005%2020_signed%20(1).pdf

Continued on next page

Report-only survey details, cont.					
**Country**	**ISO3**	**Survey Years**	**Source**	**Report Page**	**Link**
Togo	TGO	2006	MICS3	7	https://mics-surveys-prod.s3.amazonaws.com/MICS3/West%20and%20Central%20Africa/Togo/2006/Final/Togo%202006%20MICS_French.pdf
Zambia	ZMB	2008	MIS	8	https://www.malariasurveys.org/documents/Zambia_MIS_2008.pdf
Zambia	ZMB	2010	MIS	9	https://www.malariasurveys.org/documents/Zambia_MIS_2010.pdf
Zambia	ZMB	2012	MIS	8	https://www.malariasurveys.org/documents/Zambia_MIS_2012.pdf
Zambia	ZMB	2015	MIS	8	https://static1.squarespace.com/static/58d002f017bffcf99fe21889/t/5e348f9c95862d328a90cf42/1580502944434/Zambia-MIS2015_Jan20-nosigs.pdf

Continued on next page

Report-only survey details, cont.					
**Country**	**ISO3**	**Survey Years**	**Source**	**Report Page**	**Link**
Zambia	ZMB	2018	MIS	18	https://www.malariasurveys.org/documents/Zambia_MIS2018_FINAL-withSigs_20190415.pdf
Zimbabwe	ZWE	2009	MICS3	10	https://mics-surveys-prod.s3.amazonaws.com/MICS3/Eastern%20and%20Southern%20Africa/Zimbabwe/2009/Final/Zimbabwe%202009%20MICS_English.pdf

Supplementary Table 3.1: Sources for those surveys used in ITN models in which only report-level data was available.

3.2 Sensitivity Analysis Full Results

These plots show model fits for the stock and flow sensitivity analysis described in Supplement 1.6. Blue curves represent LLIN model estimates, while red curves represent cITN model estimates. Solid curves show mean values, and shaded areas represent 95% confidence intervals. Mean values for survey data is shown in circles for LLINs and squares for cITNs,with vertical lines showing the standard error of the survey. Surveys used for model fitting are shown in color, while out-of-sample surveys are shown in black. The RMSEs printed on each plot are for LLINs only.

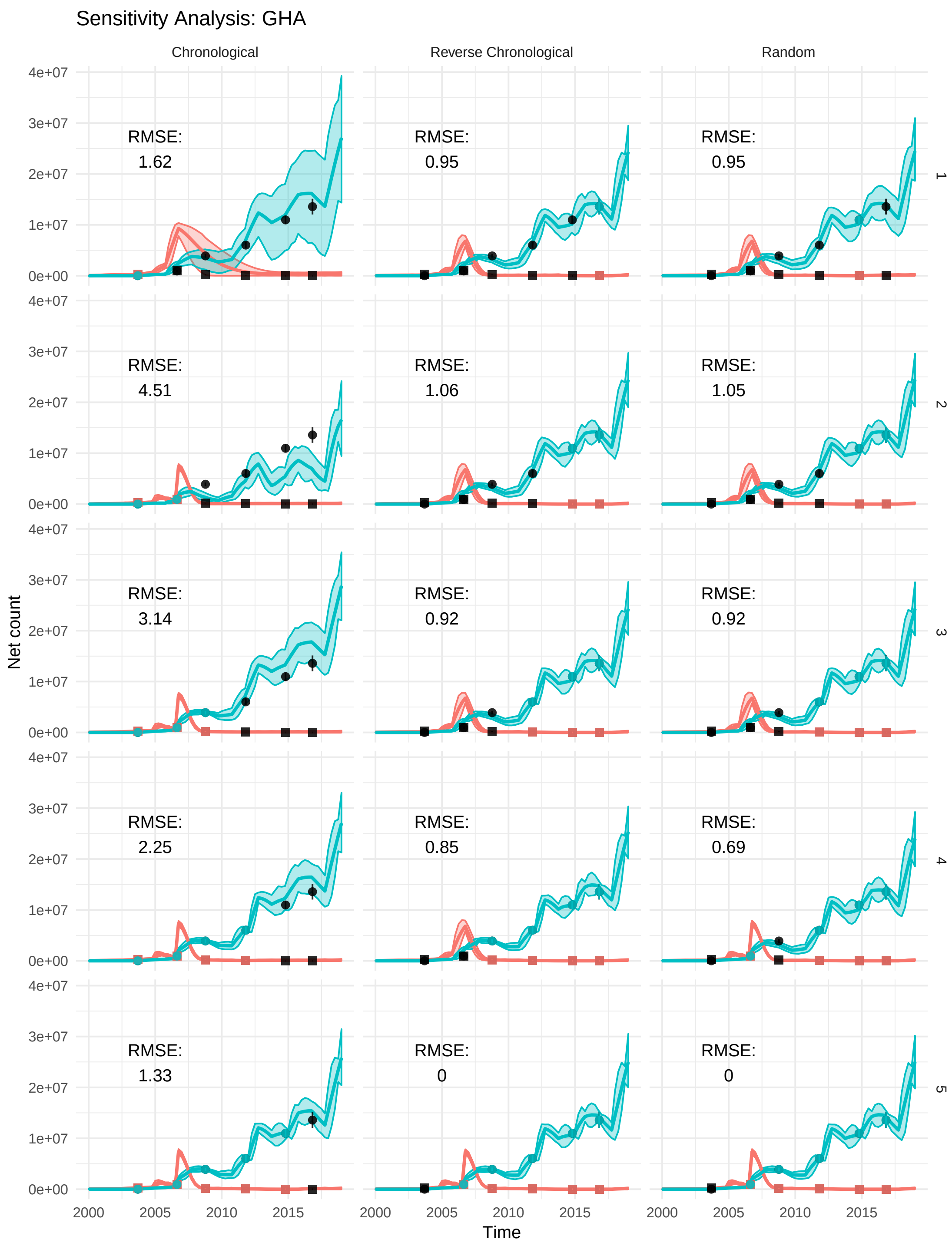

Supplementary Figure 3.1: Sensitivity analysis results for Ghana.

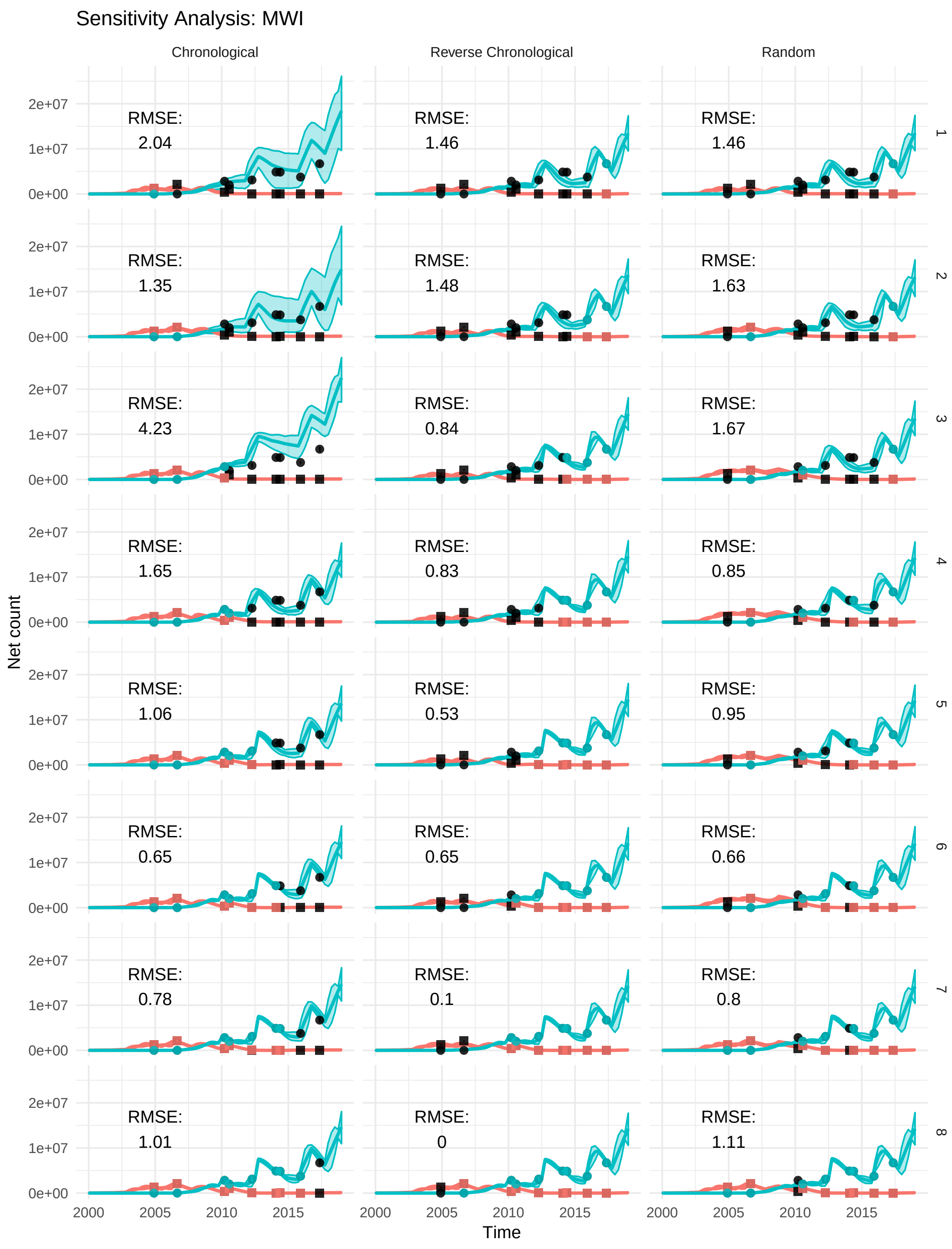

Supplementary Figure 3.2: Sensitivity analysis results for Malawi.

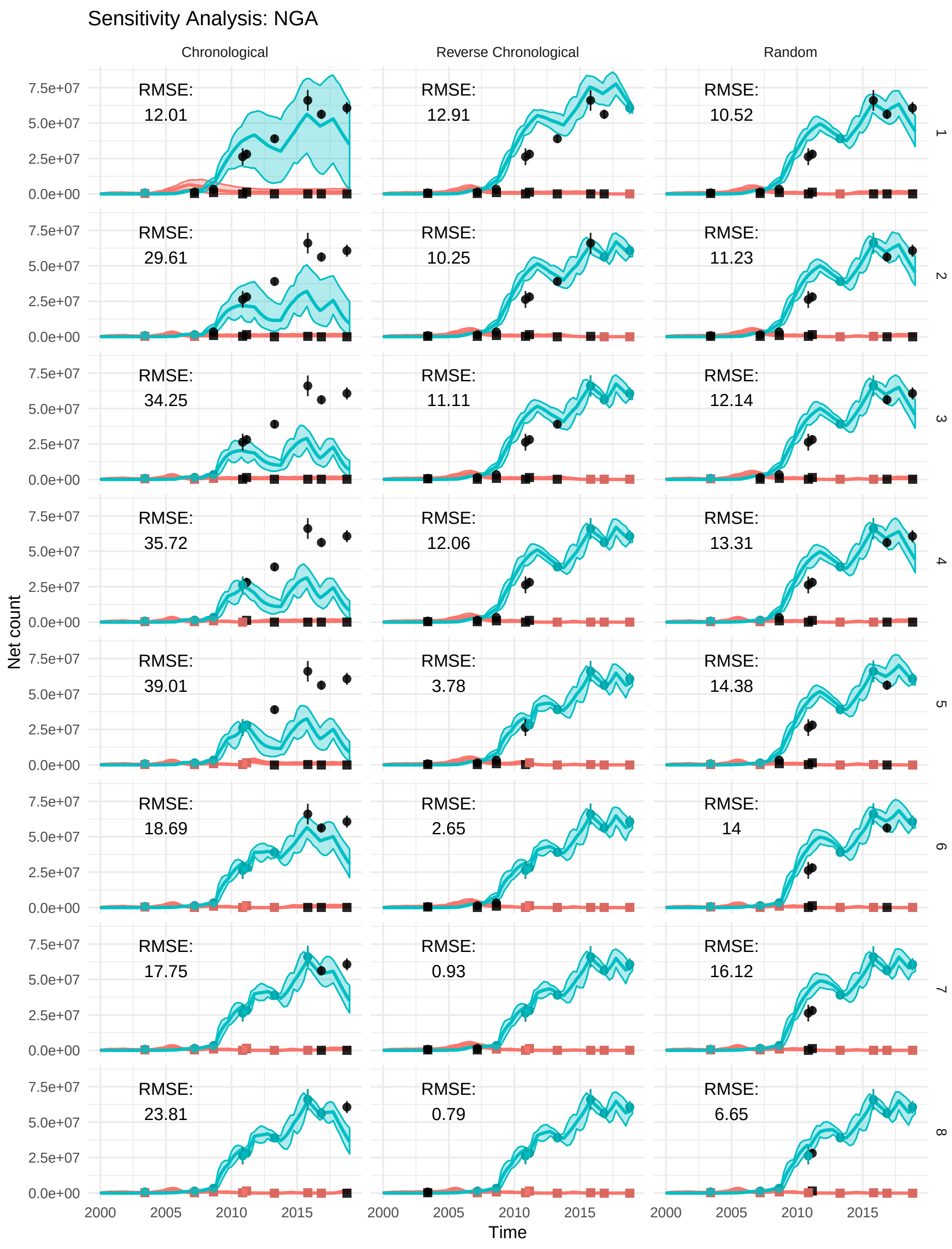

Supplementary Figure 3.3: Sensitivity analysis results for Nigeria.

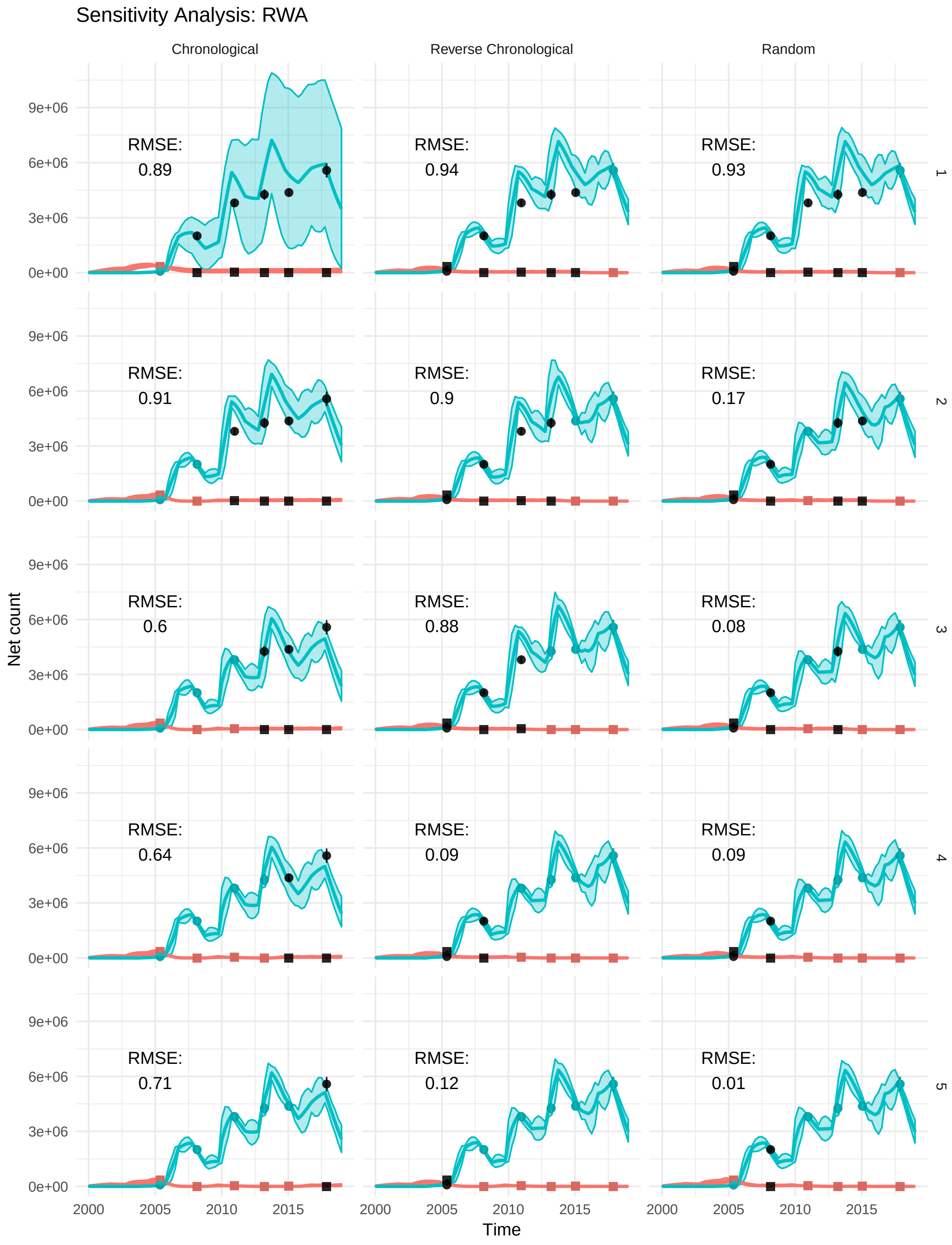

Supplementary Figure 3.4: Sensitivity analysis results for Rwanda.

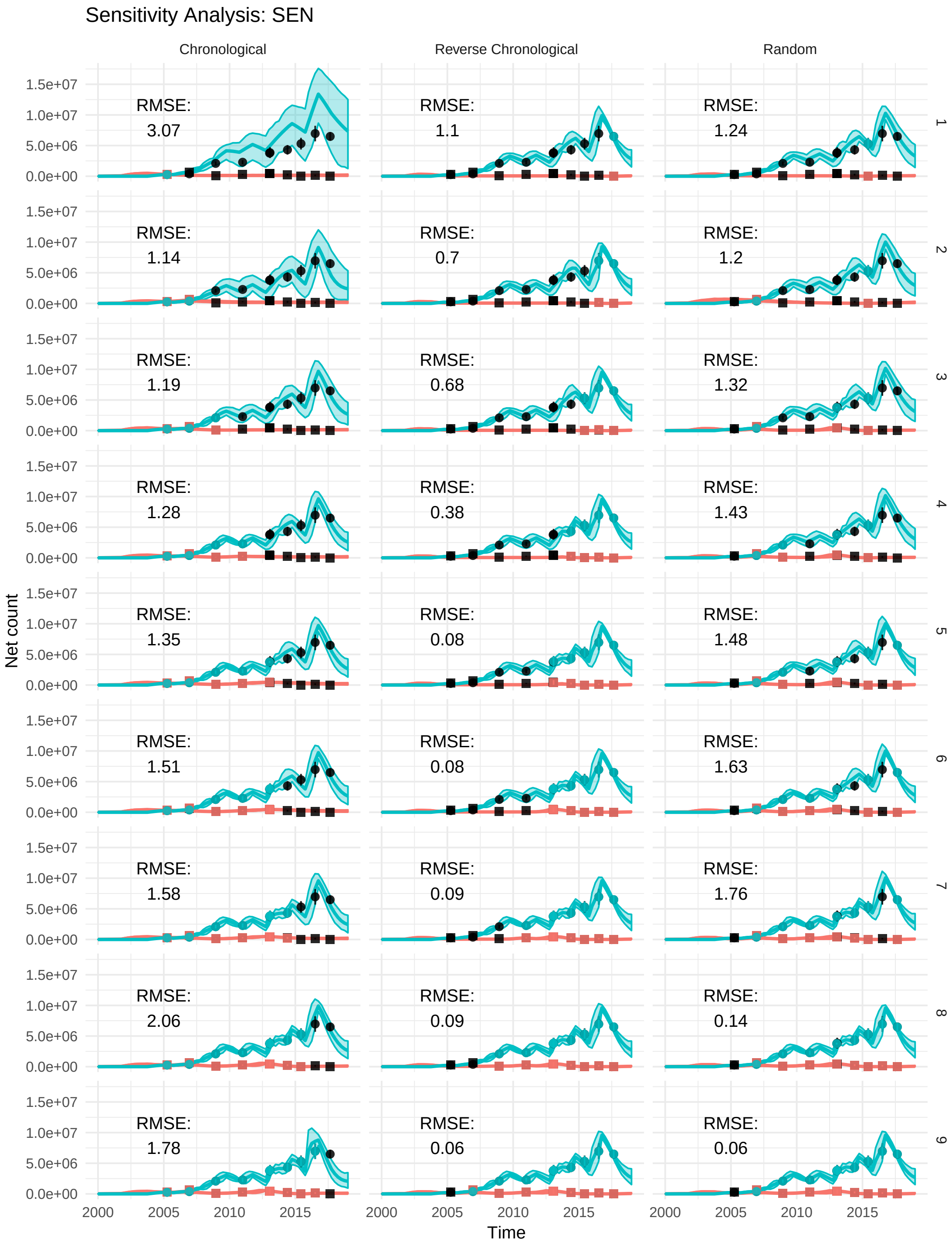

Supplementary Figure 3.5: Sensitivity analysis results for Senegal.

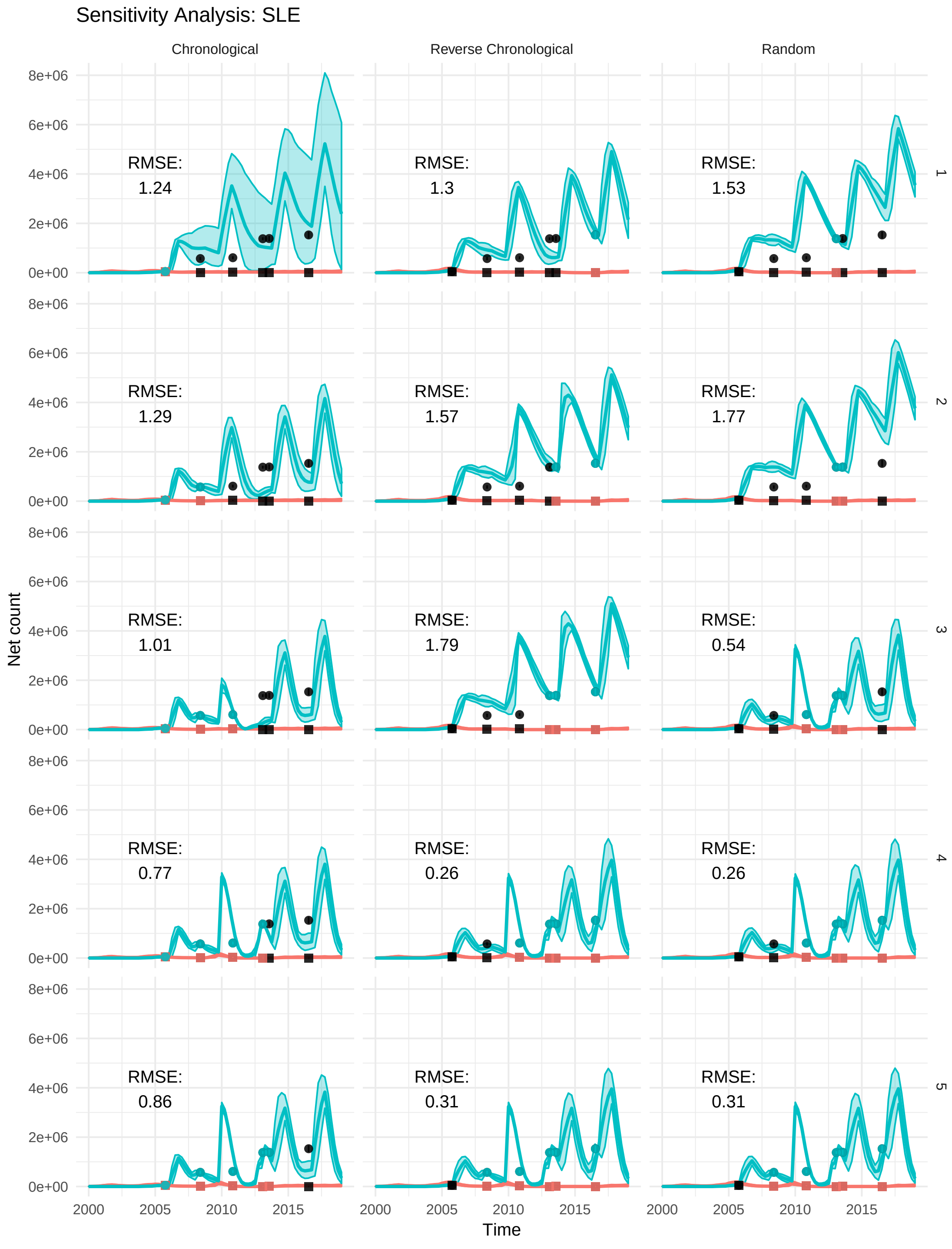

Supplementary Figure 3.6: Sensitivity analysis results for Sierra Leone.

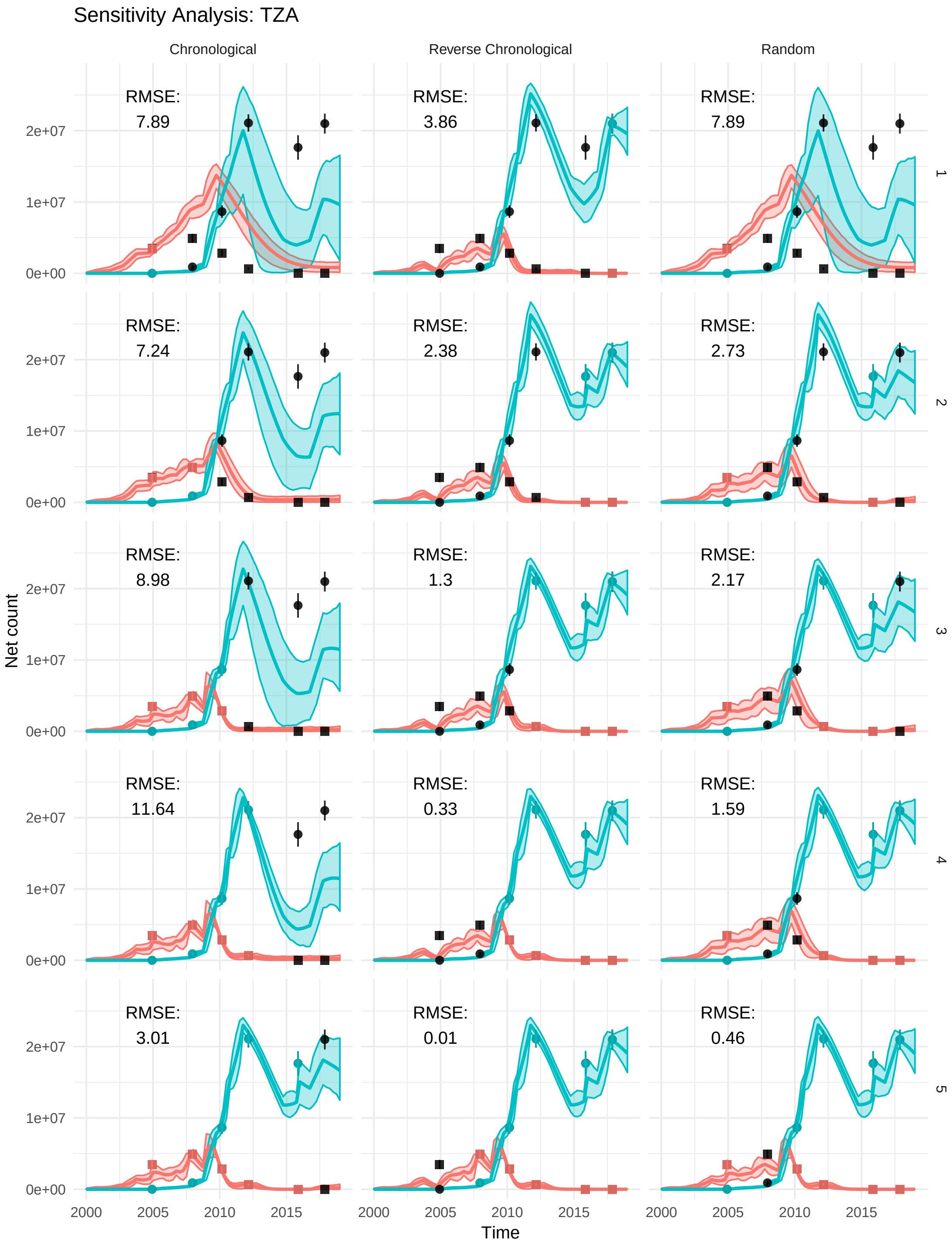

Supplementary Figure 3.7: Sensitivity analysis results for Tanzania.

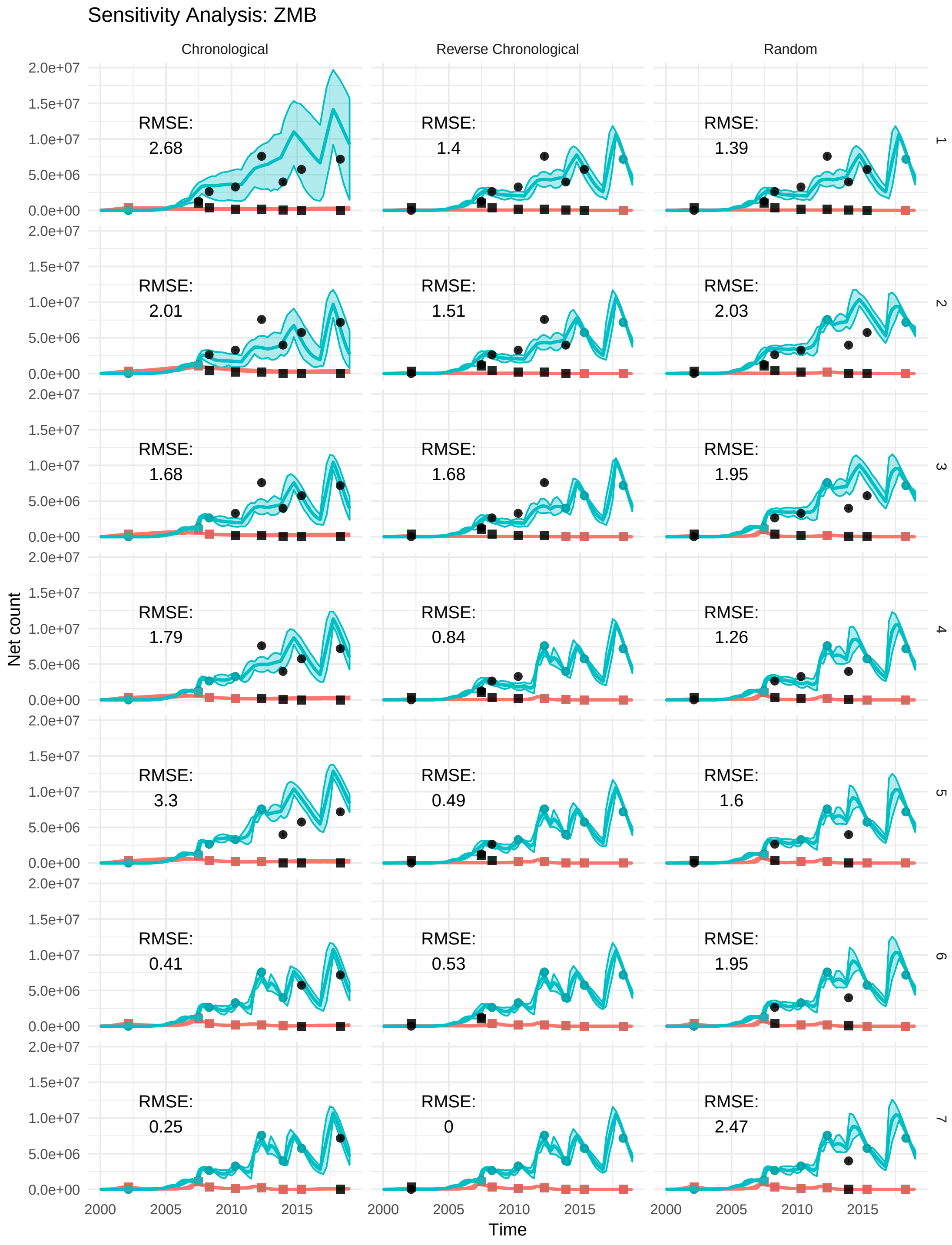

Supplementary Figure 3.8: Sensitivity analysis results for Zambia.

3.3 LLIN Retention Caterpillar Plots

These plots show posterior densities and parameter traces from JAGS for the LLIN duration parameter in the stock and flow model (defined as τ in Supplementary Table 1.3). Countries with wide densities or trace plots that cluster toward the top or bottom of the plot suggest unstable model fits, usually due to sparse survey data.

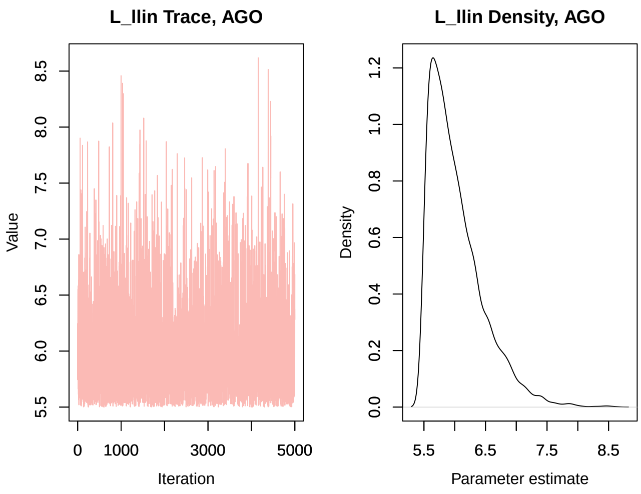

Supplementary Figure 3.9: Posterior density and parameter trace plots for Angola.

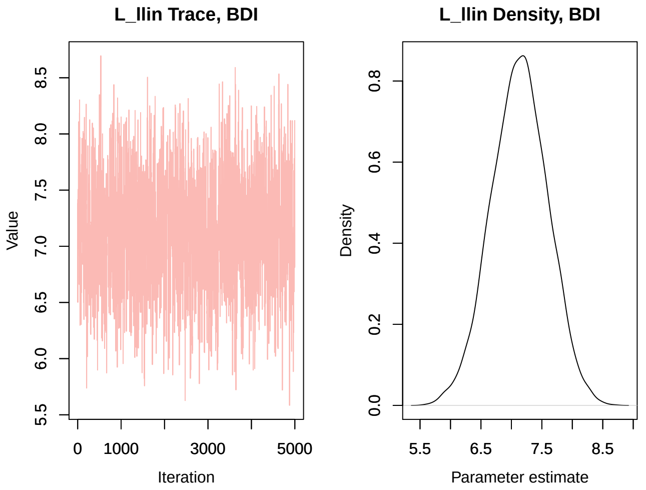

Supplementary Figure 3.10: Posterior density and parameter trace plots for Burundi.

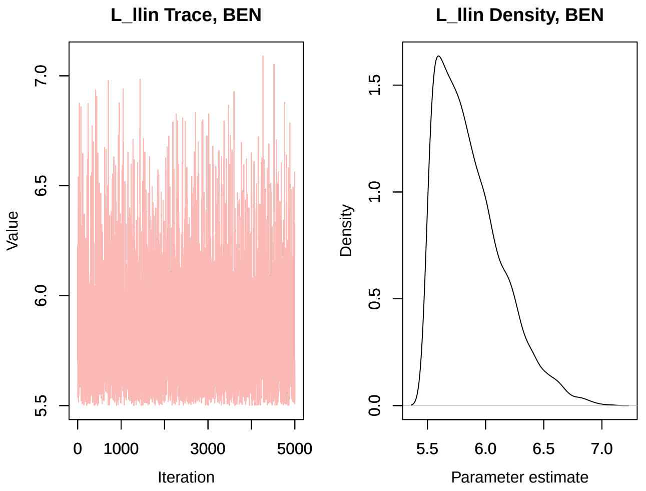

Supplementary Figure 3.11: Posterior density and parameter trace plots for Benin.

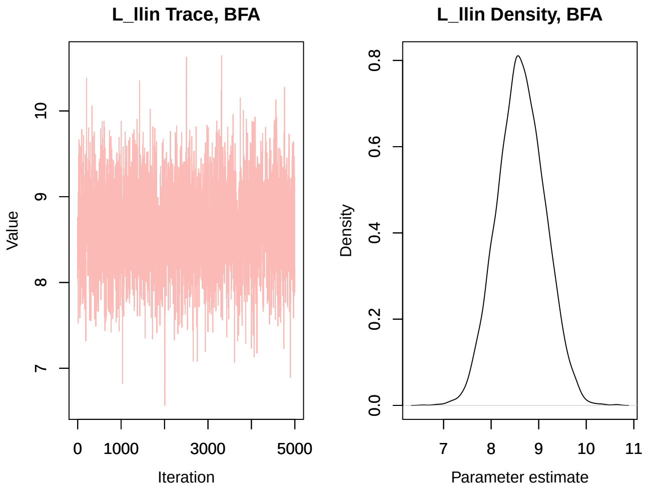

Supplementary Figure 3.12: Posterior density and parameter trace plots for Burkina Faso.

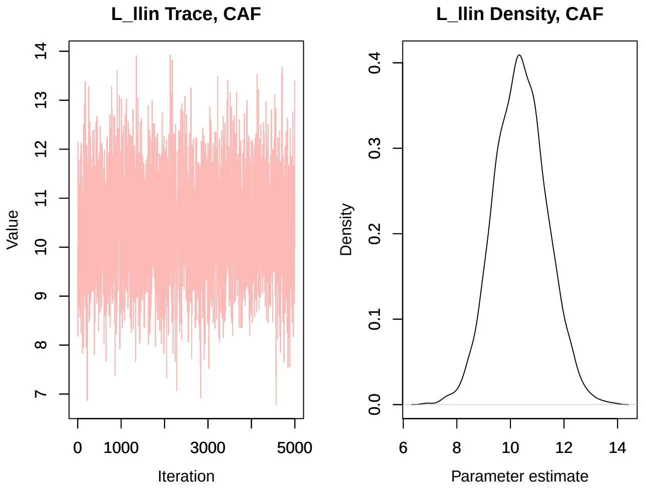

Supplementary Figure 3.13: Posterior density and parameter trace plots for Central African Republic.

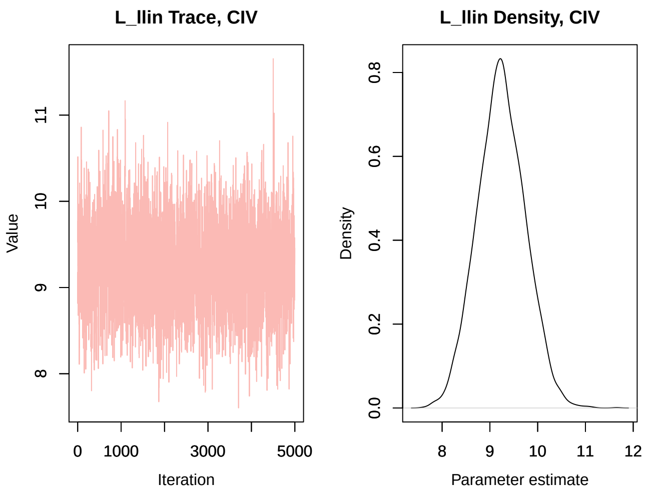

Supplementary Figure 3.14: Posterior density and parameter trace plots for Cote d'Ivoire.

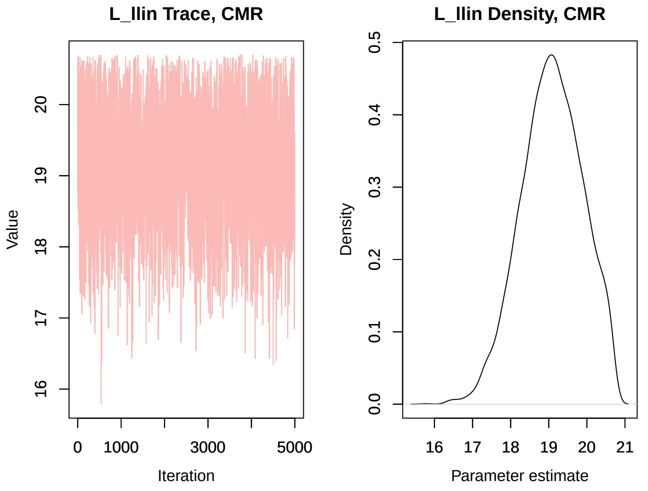

Supplementary Figure 3.15: Posterior density and parameter trace plots for Cameroon.

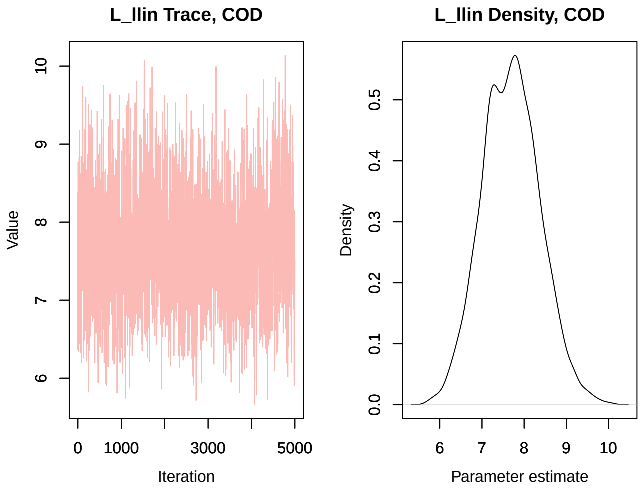

Supplementary Figure 3.16: Posterior density and parameter trace plots for Democratic Republic of the Congo.

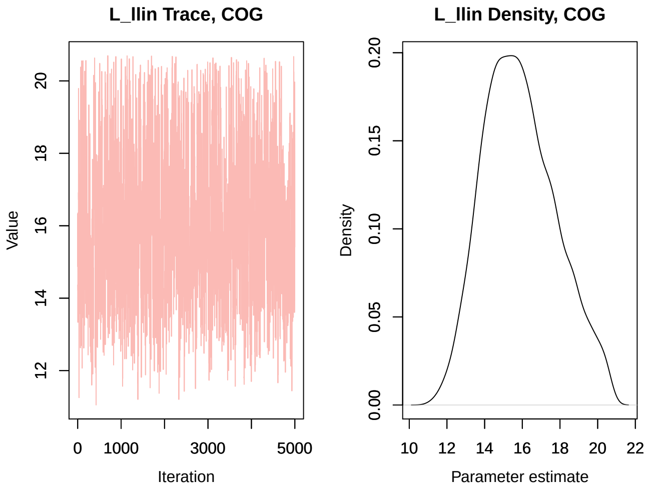

Supplementary Figure 3.17: Posterior density and parameter trace plots for Republic of Congo.

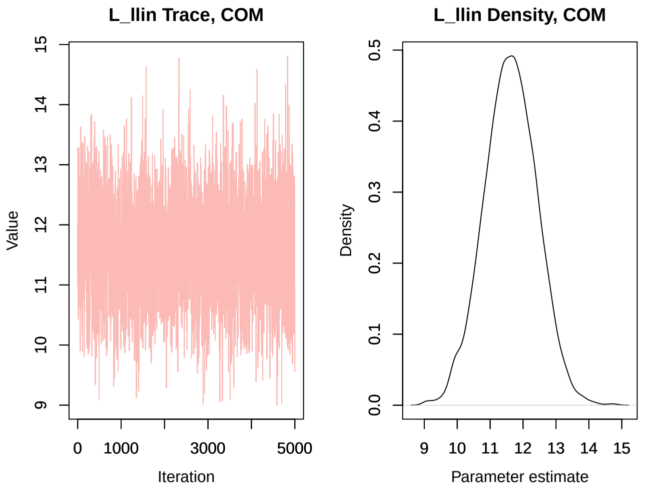

Supplementary Figure 3.18: Posterior density and parameter trace plots for Comoros.

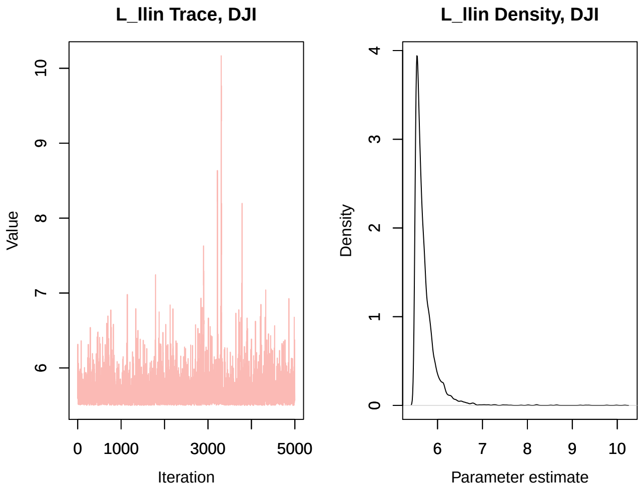

Supplementary Figure 3.19: Posterior density and parameter trace plots for Djibouti.

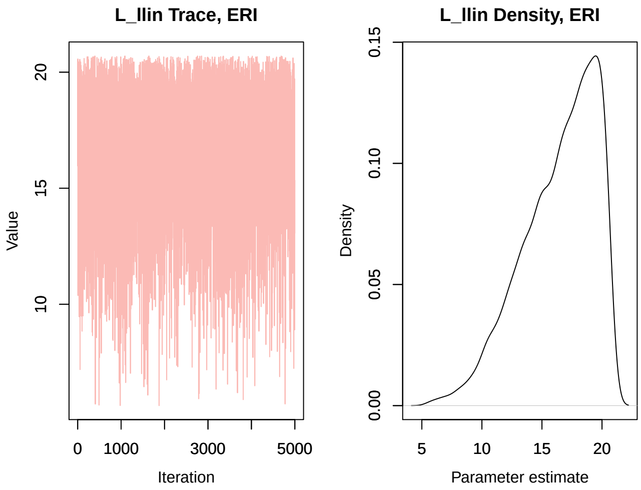

Supplementary Figure 3.20: Posterior density and parameter trace plots for Eritrea.

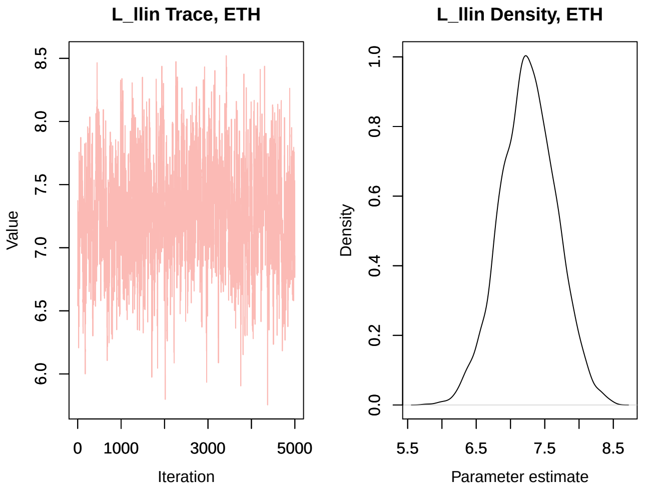

Supplementary Figure 3.21: Posterior density and parameter trace plots for Ethiopia.

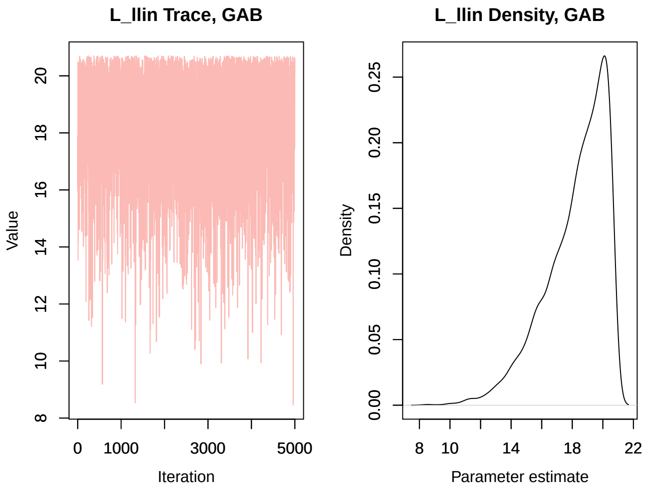

Supplementary Figure 3.22: Posterior density and parameter trace plots for Gabon.

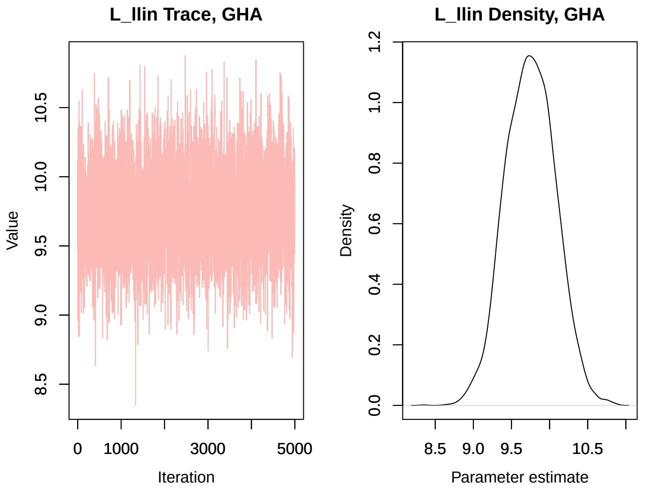

Supplementary Figure 3.23: Posterior density and parameter trace plots for Ghana.

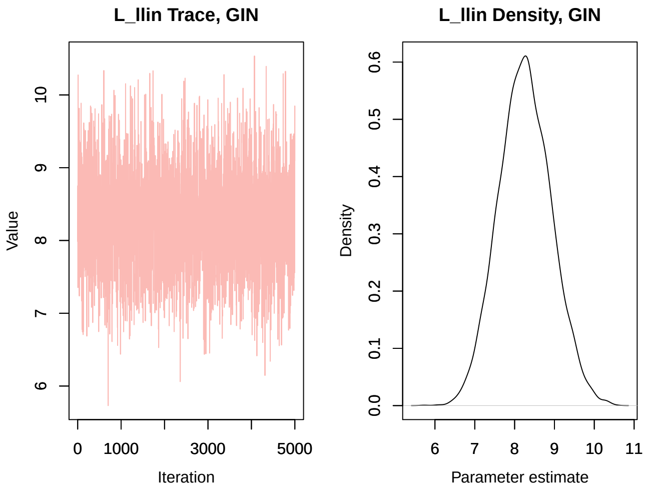

Supplementary Figure 3.24: Posterior density and parameter trace plots for Guinea.

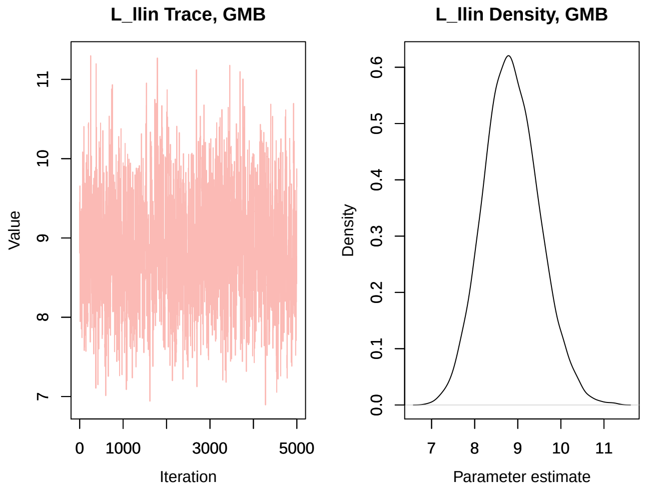

Supplementary Figure 3.25: Posterior density and parameter trace plots for Gambia.

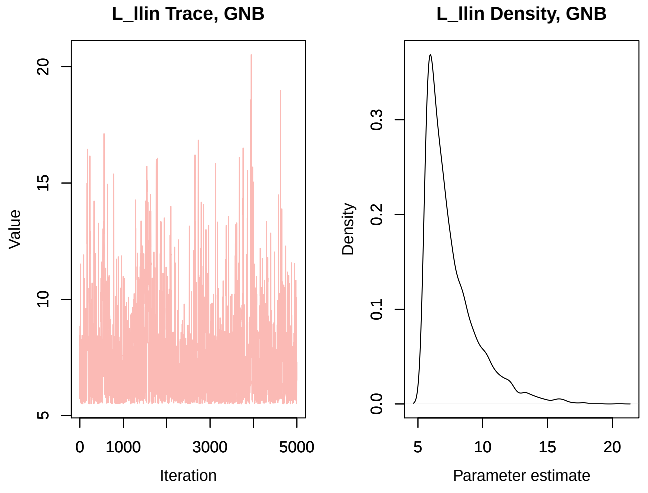

Supplementary Figure 3.26: Posterior density and parameter trace plots for Guinea-Bissau.

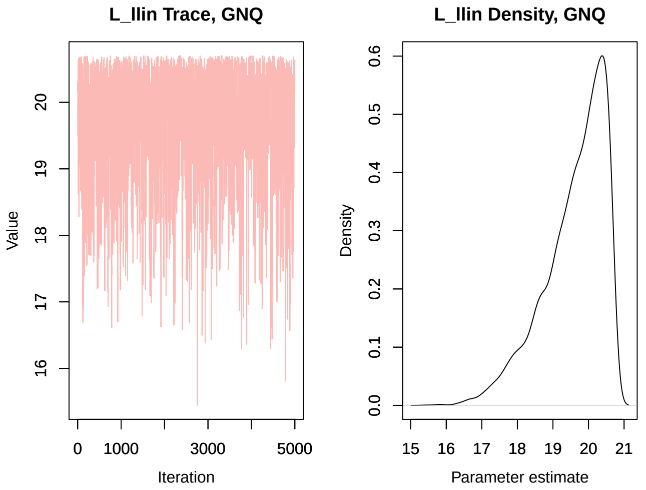

Supplementary Figure 3.27: Posterior density and parameter trace plots for Equatorial Guinea.

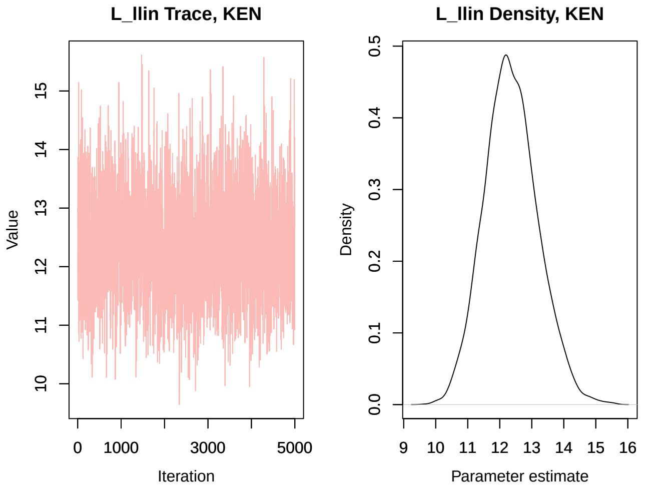

Supplementary Figure 3.28: Posterior density and parameter trace plots for Kenya.

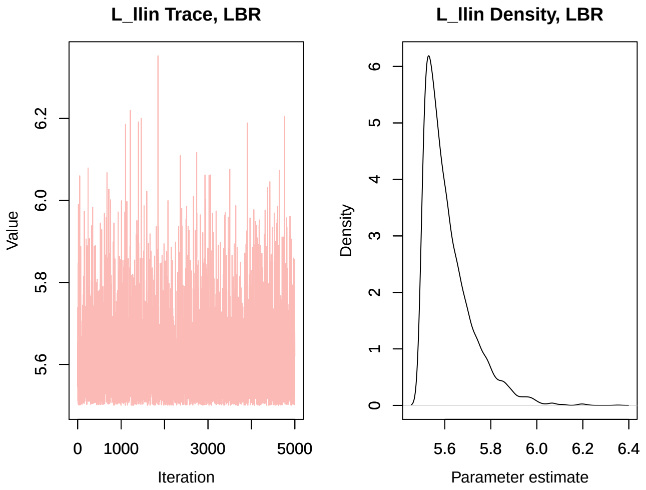

Supplementary Figure 3.29: Posterior density and parameter trace plots for Liberia.

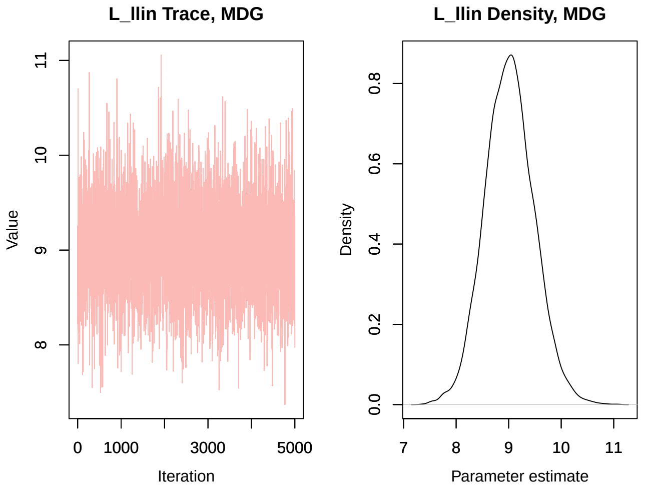

Supplementary Figure 3.30: Posterior density and parameter trace plots for Madagascar.

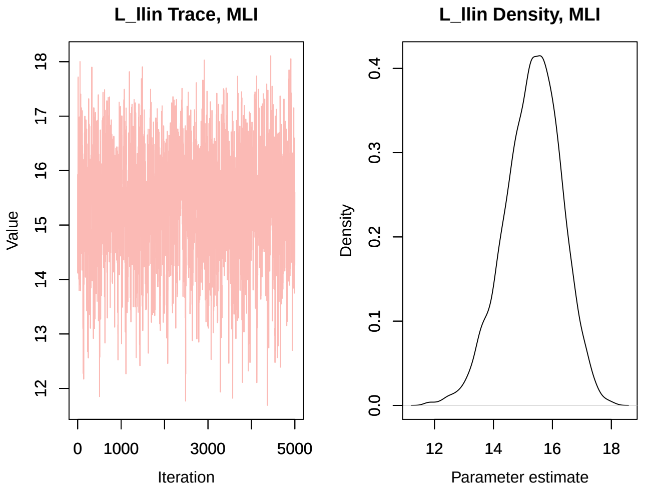

Supplementary Figure 3.31: Posterior density and parameter trace plots for Mali.

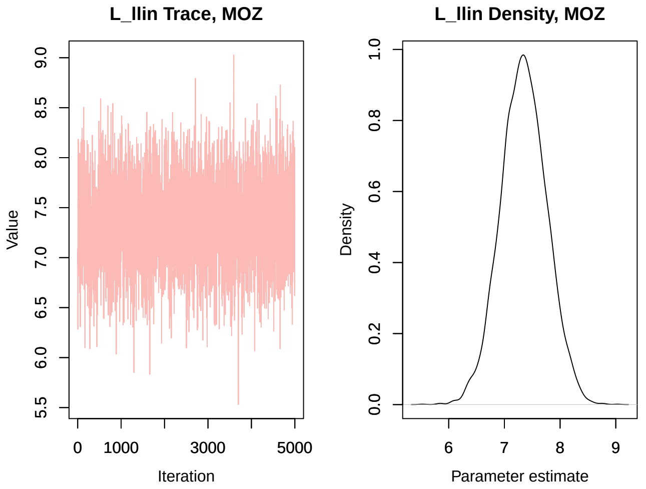

Supplementary Figure 3.32: Posterior density and parameter trace plots for Mozambique.

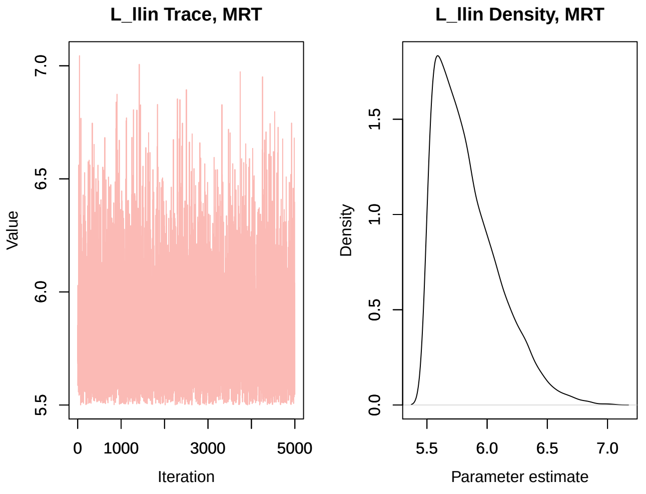

Supplementary Figure 3.33: Posterior density and parameter trace plots for Mauritania.

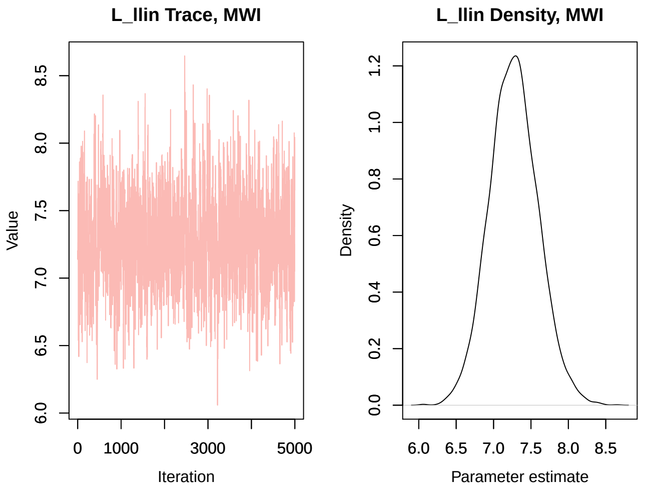

Supplementary Figure 3.34: Posterior density and parameter trace plots for Malawi.

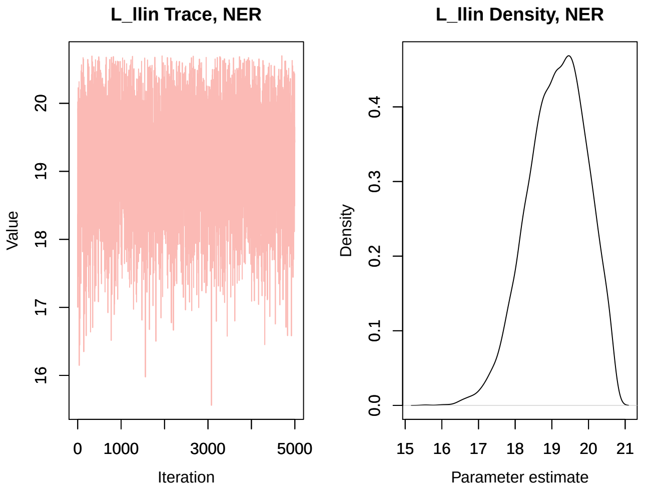

Supplementary Figure 3.35: Posterior density and parameter trace plots for Niger.

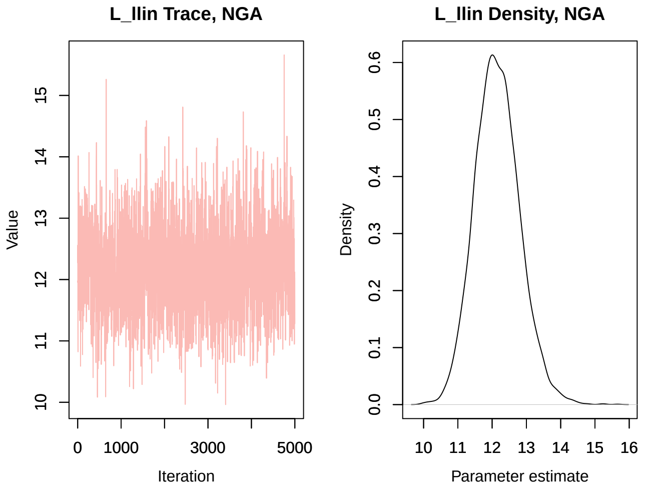

Supplementary Figure 3.36: Posterior density and parameter trace plots for Nigeria.

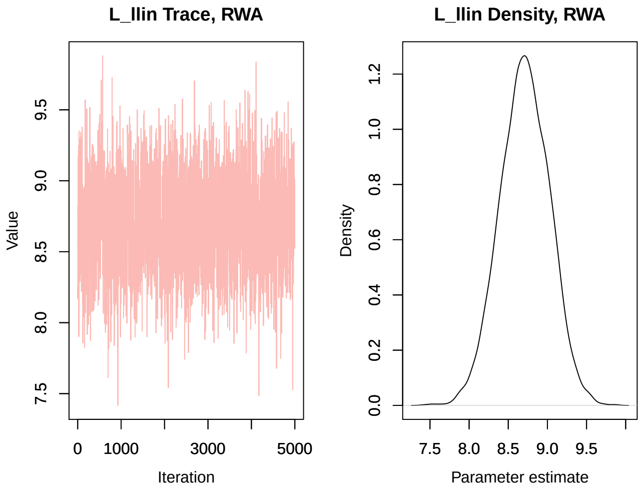

Supplementary Figure 3.37: Posterior density and parameter trace plots for Rwanda.

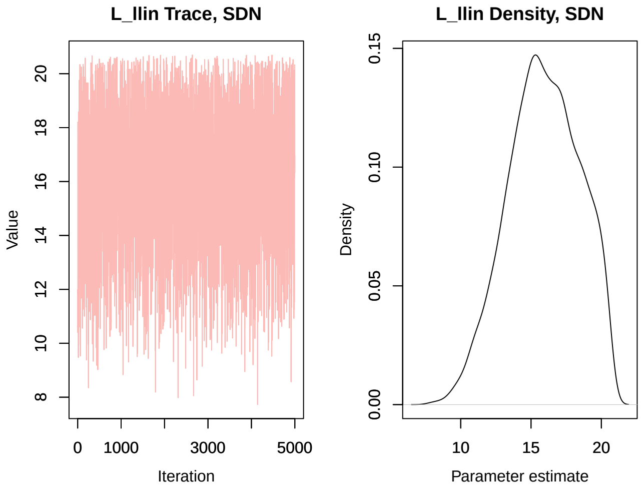

Supplementary Figure 3.38: Posterior density and parameter trace plots for Sudan.

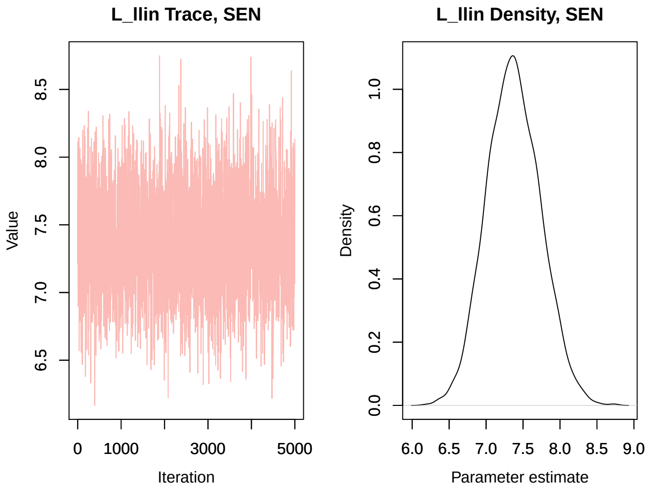

Supplementary Figure 3.39: Posterior density and parameter trace plots for Senegal.

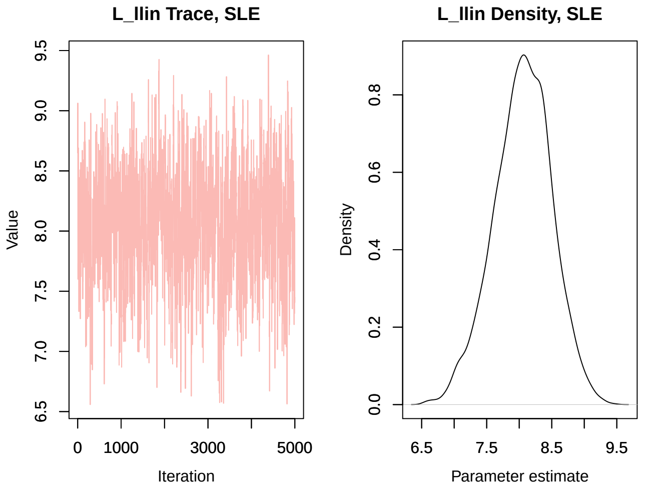

Supplementary Figure 3.40: Posterior density and parameter trace plots for Sierra Leone.

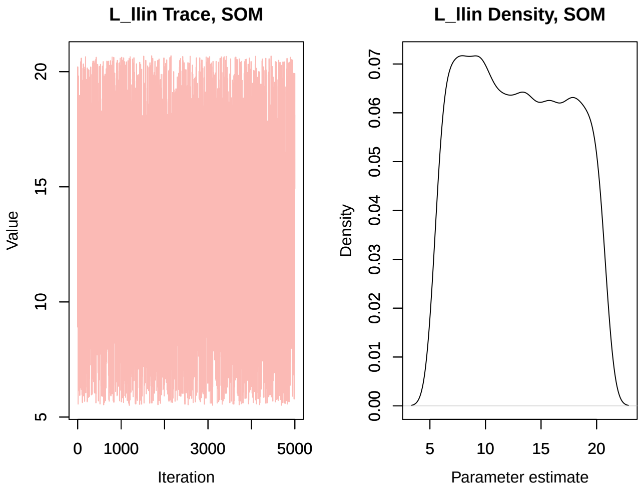

Supplementary Figure 3.41: Posterior density and parameter trace plots for Somalia.

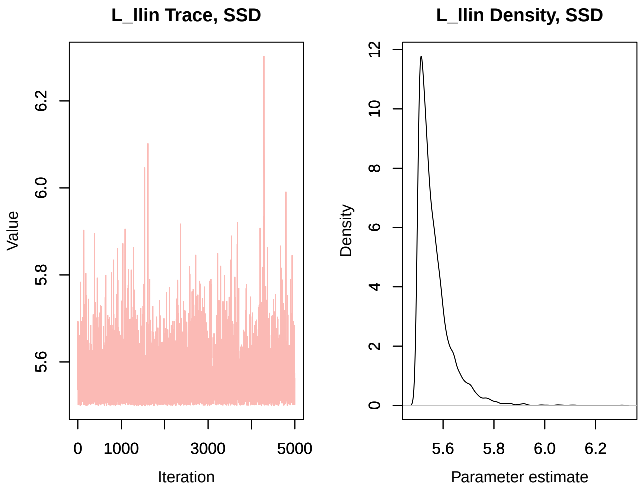

Supplementary Figure 3.42: Posterior density and parameter trace plots for South Sudan.

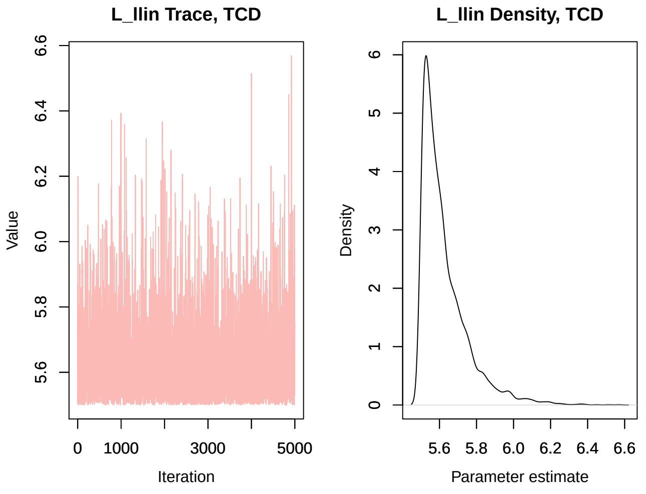

Supplementary Figure 3.43: Posterior density and parameter trace plots for Chad.

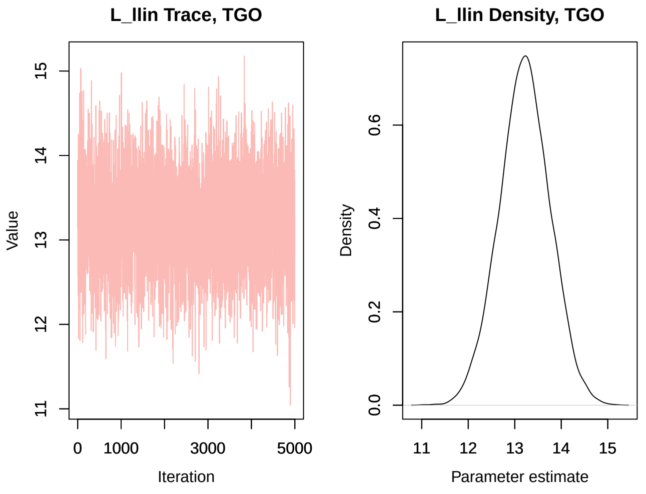

Supplementary Figure 3.44: Posterior density and parameter trace plots for Togo.

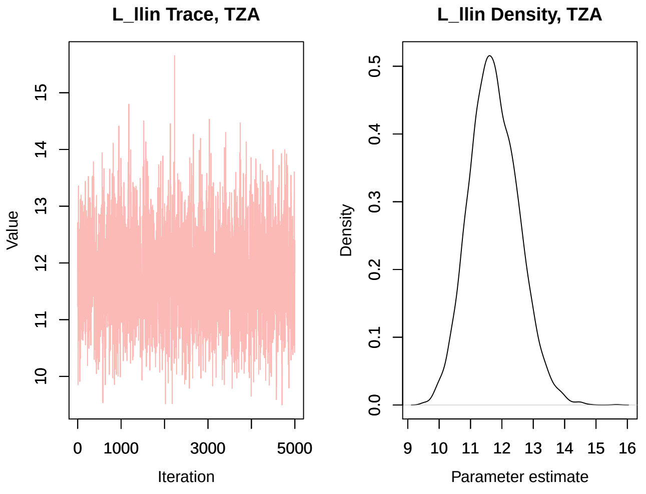

Supplementary Figure 3.45: Posterior density and parameter trace plots for Tanzania.

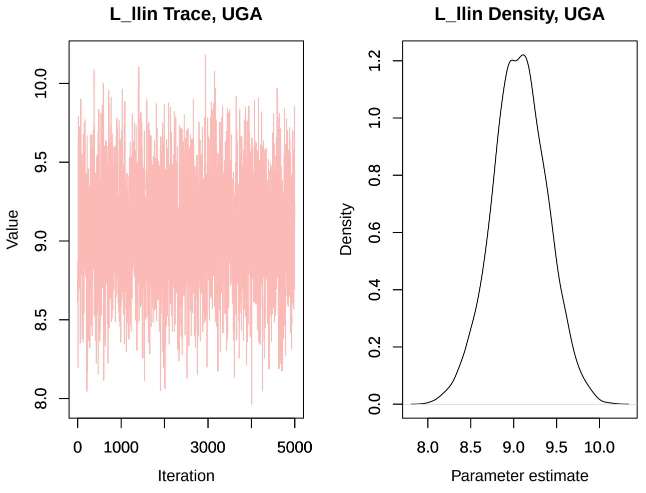

Supplementary Figure 3.46: Posterior density and parameter trace plots for Uganda.

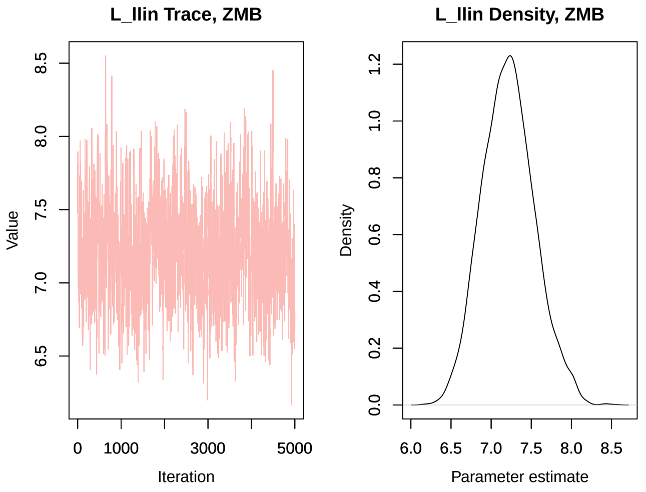

Supplementary Figure 3.47: Posterior density and parameter trace plots for Zambia.

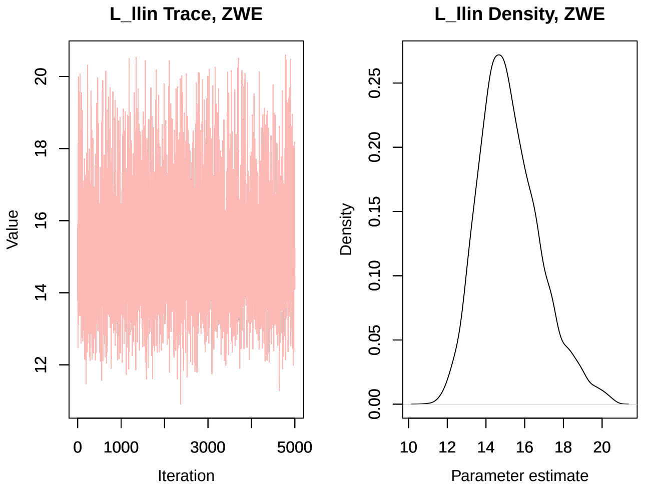

Supplementary Figure 3.48: Posterior density and parameter trace plots for Zimbabwe.

Supplementary References

1. Flaxman, A. D. *et al.* Rapid scaling up of insecticide-treated bed net coverage in Africa and its relationship with development assistance for health: A systematic synthesis of supply, distribution, and household survey data. *PLoS Medicine* **7.** ISSN: 15491277 (2010).
2. Bhatt, S. *et al.* Coverage and system efficiencies of insecticide-treated nets in Africa from 2000 to 2017. *eLife* **4.** ISSN: 2050084X (2015).
3. Yukich, J. *et al.* Planning long lasting insecticide treated net campaigns: Should households' existing nets be taken into account? *Parasites and Vectors* **6,** 174. ISSN: 17563305. https://parasitesandvectors.biomedcentral.com/articles/10.1186/1756-3305-6-174 (2013).
4. Hakizimana, E. *et al.* Monitoring long-lasting insecticidal net (LLIN) durability to validate net serviceable life assumptions, in Rwanda. *Malaria Journal* **13,** 344. ISSN: 14752875. http://malariajournal.biomedcentral.com/articles/10.1186/1475-2875-13-344 (2014).
5. Gnanguenon, V., Azondekon, R., Oke-Agbo, F., Beach, R. & Akogbeto, M. Durability assessment results suggest a serviceable life of two, rather than three, years for the current long-lasting insecticidal (mosquito) net (LLIN) intervention in Benin. *BMC Infectious Diseases* **14,** 69. ISSN: 14712334. https://bmcinfectdis.biomedcentral.com/articles/10.1186/1471-2334-14-69 (2014).
6. Solomon, T. *et al.* Bed nets used to protect against malaria do not last long in a semi-arid area of Ethiopia: A cohort study. *Malaria Journal* **17,** 239. ISSN: 14752875. https://malariajournal.biomedcentral.com/articles/10.1186/s12936-018-2391-5 (2018).
7. Weiss, D. J. *et al.* Mapping the global prevalence, incidence, and mortality of Plasmodium falciparum, 2000–17: a spatial and temporal modelling study. *The Lancet* **394,** 322–331. ISSN: 1474547X (2019).
8. Kilian, A., Boulay, M., Koenker, H. & Lynch, M. How many mosquito nets are needed to achieve universal coverage? Recommendations for the quantification and allocation of long-lasting insecticidal nets for mass campaigns. *Malaria Journal* **9,** 330. ISSN: 14752875. https://malariajournal.biomedcentral.com/articles/10.1186/1475-2875-9-330 (2010).
9. Singh, J. A Characterization of Positive Poisson Distribution and its Statistical Application. *SIAM Journal on Applied Mathematics* **34,** 545–548 (1978).
10. Bhatt, S. *et al.* The effect of malaria control on Plasmodium falciparum in Africa between 2000 and 2015. *Nature* **526,** 207–211. ISSN: 14764687 (2015).

11. Weiss, D. J. *et al.* An effective approach for gap-filling continental scale remotely sensed time-series. *ISPRS Journal of Photogrammetry and Remote Sensing* **98,** 106–118. ISSN: 09242716. /pmc/articles/PMC4308023/?report=abstract%20https://www.ncbi.nlm.nih.gov/pmc/articles/PMC4308023/ (2014).

12. Lindgren, F., Rue, H. & Lindström, J. An explicit link between gaussian fields and gaussian markov random fields: The stochastic partial differential equation approach. *Journal of the Royal Statistical Society. Series B: Statistical Methodology* **73,** 423–498. ISSN: 13697412. https://rss.onlinelibrary.wiley.com/doi/full/10.1111/j.1467-9868.2011.00777.x%20https://rss.onlinelibrary.wiley.com/doi/abs/10.1111/j.1467-9868.2011.00777.x%20https://rss.onlinelibrary.wiley.com/doi/10.1111/j.1467-9868.2011.00777.x (2011).

13. Burbidge, J. B., Magee, L. & Robb, A. L. Alternative Transformations to Handle Extreme Values of the Dependent Variable. *Journal of the American Statistical Association* **83,** 123. ISSN: 01621459 (1988).

14. Koenker, H. *et al.* Quantifying seasonal variation in insecticide-treated net use among those with access. *American Journal of Tropical Medicine and Hygiene* **101,** 371–382. ISSN: 00029637 (2019).

15. Winch, P. J. *et al.* Seasonal variation in the perceived risk of malaria: Implications for the promotion of insecticide-impregnated bed nets. *Social Science and Medicine* **39,** 63–75. ISSN: 02779536 (1994).

16. Battle, K. E. *et al.* Mapping the global endemicity and clinical burden of Plasmodium vivax, 2000–17: a spatial and temporal modelling study. *The Lancet* **394,** 332–343. ISSN: 1474547X. http://dx.doi.org/10.1016/ (2019).
