# Supplementary material for: Maps and metrics of insecticide-treated net access, use, and nets-per-capita in Africa from 2000-2020
Source: Nat Commun. 2021 Jun 11;12:3589. doi: 10.1038/s41467-021-23707-7 (PMC8196080; doi:10.1038/s41467-021-23707-7)
Supplement: Supplementary file 3 — Reporting Summary [file 41467_2021_23707_MOESM3_ESM.pdf]

nature research

Corresponding author(s): Amelia Bertozzi-Villa

Last updated by author(s): May 7, 2021

Reporting Summary

Nature Research wishes to improve the reproducibility of the work that we publish. This form provides structure for consistency and transparency in reporting. For further information on Nature Research policies, see our Editorial Policies and the Editorial Policy Checklist.

Statistics

For all statistical analyses, confirm that the following items are present in the figure legend, table legend, main text, or Methods section.

n/a	Confirmed	
☐	☒	The exact sample size (*n*) for each experimental group/condition, given as a discrete number and unit of measurement
☒	☐	A statement on whether measurements were taken from distinct samples or whether the same sample was measured repeatedly
☐	☒	The statistical test(s) used AND whether they are one- or two-sided<br>*Only common tests should be described solely by name; describe more complex techniques in the Methods section.*
☐	☒	A description of all covariates tested
☐	☒	A description of any assumptions or corrections, such as tests of normality and adjustment for multiple comparisons
☐	☒	A full description of the statistical parameters including central tendency (e.g. means) or other basic estimates (e.g. regression coefficient) AND variation (e.g. standard deviation) or associated estimates of uncertainty (e.g. confidence intervals)
☒	☐	For null hypothesis testing, the test statistic (e.g. *F*, *t*, *r*) with confidence intervals, effect sizes, degrees of freedom and *P* value noted<br>*Give P values as exact values whenever suitable.*
☐	☒	For Bayesian analysis, information on the choice of priors and Markov chain Monte Carlo settings
☒	☐	For hierarchical and complex designs, identification of the appropriate level for tests and full reporting of outcomes
☒	☐	Estimates of effect sizes (e.g. Cohen's *d*, Pearson's *r*), indicating how they were calculated

Our web collection on statistics for biologists contains articles on many of the points above.

Software and code

Policy information about availability of computer code

Data collection	Data from DHS was downloaded from https://dhsprogram.com/ using the code in https://github.com/harry-gibson/DHS-To-Database, and the ITN-relevant variables selected using the SQL queries available at https://github.com/harry-gibson/DHS-Data-Extractions/tree/main/ITN_Access_and_Use/SQL. Data from MICS surveys, MIS reports, net manufacturer deliveries, and NMCP distributions of nets was collected manually and cleaned using the code described in the ``Data Analysis'' section. No additional software was used for data collection.
Data analysis	All code used for data analysis and modeling is publicly available in the "publication-2021" branch of "https://github.com/bertozzivill/map-itn-cube/tree/publication-2021", and in the corresponding release labeled "nat-comms-submission". This repository also contains the data and code used to generate the figures in this paper. For analyses run on Google Cloud, Dockerfiles can be shared upon request. This analysis was run using R version 3.6.3, INLA version 20.03.17, rjags version 4.3.0, and rstan version 2.19.3.

For manuscripts utilizing custom algorithms or software that are central to the research but not yet described in published literature, software must be made available to editors and reviewers. We strongly encourage code deposition in a community repository (e.g. GitHub). See the Nature Research guidelines for submitting code & software for further information.

Data

Policy information about availability of data

All manuscripts must include a data availability statement. This statement should provide the following information, where applicable:
- Accession codes, unique identifiers, or web links for publicly available datasets
- A list of figures that have associated raw data
- A description of any restrictions on data availability

The household-level survey data used in this analysis is publicly available from the DHS (https://dhsprogram.com/) and MICS (https://mics.unicef.org/) websites. The national-level aggregated survey data was gleaned from reports available at the MIS website (https://www.malariasurveys.org/, see Supplementary Table 3.1 for

links to specific reports). Data on manufacturer delivery of nets is available from the AMP Net Mapping Project (https://allianceformalariaprevention.com/net-mapping-project/). Data on NMCP distribution of nets will be available via WHO and from ALMA in the coming months. Prospective distribution estimates from PMI reports are available at https://www.pmi.gov/resource-library/mops/fy-2020. All covariate data is available from the sources listed in Supplementary Table 2.1, and the specific versions used in this analysis can be found at https://malariaatlas.org/research-project/metrics-of-insecticide-treated-nets-distribution.

All results, including annual rasters of ITN access, use, use rate, and nets-per-capita with upper and lower bounds, are available at https://malariaatlas.org/research-project/metrics-of-insecticide-treated-nets-distribution. This page also contains an interactive uncertainty visualization, access to the covariate data used in this analysis, and other helpful links. Data used in the figures of this publication can be found, along with plotting code, at the GitHub repository in the Code Availability section.

Field-specific reporting

Please select the one below that is the best fit for your research. If you are not sure, read the appropriate sections before making your selection.

☐ Life sciences ☒ Behavioural & social sciences ☐ Ecological, evolutionary & environmental sciences

For a reference copy of the document with all sections, see nature.com/documents/nr-reporting-summary-flat.pdf

Behavioural & social sciences study design

All studies must disclose on these points even when the disclosure is negative.

Study description	This is a secondary data analysis that utilized previously-collected and publicly-available survey data. Details on the structure of each individual survey can be found at their respective websites (listed in the data availability statement above).
Research sample	This parameter is not relevant to this analysis because we utilized only secondary data.
Sampling strategy	This parameter is not relevant to this analysis because we utilized only secondary data.
Data collection	This parameter is not relevant to this analysis because we utilized only secondary data.
Timing	This parameter is not relevant to this analysis because we utilized only secondary data.
Data exclusions	This parameter is not relevant to this analysis because we utilized only secondary data.
Non-participation	This parameter is not relevant to this analysis because we utilized only secondary data.
Randomization	This parameter is not relevant to this analysis because we utilized only secondary data.

Reporting for specific materials, systems and methods

We require information from authors about some types of materials, experimental systems and methods used in many studies. Here, indicate whether each material, system or method listed is relevant to your study. If you are not sure if a list item applies to your research, read the appropriate section before selecting a response.

Materials & experimental systems

n/a	Involved in the study	
☒	☐	Antibodies
☒	☐	Eukaryotic cell lines
☒	☐	Palaeontology and archaeology
☒	☐	Animals and other organisms
☒	☐	Human research participants
☒	☐	Clinical data
☒	☐	Dual use research of concern

Methods

n/a	Involved in the study	
☒	☐	ChIP-seq
☒	☐	Flow cytometry
☒	☐	MRI-based neuroimaging
